# Supplementary material for: Psychological and pharmacological interventions for posttraumatic stress disorder and comorbid mental health problems following complex traumatic events: Systematic review and component network meta-analysis
Source: PLoS Med. 2020 Aug 19;17(8):e1003262. doi: 10.1371/journal.pmed.1003262 (PMC7446790; doi:10.1371/journal.pmed.1003262)
Supplement: S2 Text — (DOCX) [file pmed.1003262.s002.docx]

**S2 Appendix Sample search strategies**

**MEDLINE**

**Via OVID**

**Database: Ovid MEDLINE(R) Epub Ahead of Print, In-Process & Other Non-Indexed Citations, Ovid MEDLINE(R) Daily and Ovid MEDLINE(R) <1946 to Present>**

1 exp Violence/ (84653)

2 violence.ti,ab. (38287)

3 Domestic Violence/ (5769)

4 Intimate Partner Violence/ (716)

5 Battered Women/ (2579)

6 (batter$ adj2 (wife$ or wive$ or woman or women or men or husband$ or partner$)).ti,ab. (870)

7 (physical$ adj2 (abus$ or assault$ or violen$ or aggress$)).ti,ab. (10464)

8 (emotional$ adj2 abus$).ti,ab. (1749)

9 Rape/ (6157)

10 rape.ti,ab. (6600)

11 (sexual$ adj2 (abus$ or assault$ or violen$ or aggress$)).ti,ab. (19466)

12 Child Sexual Abuse/ (9551)

13 child$ sexual abuse.ti,ab. (4339)

14 (child$ adj2 exploit$).ti,ab. (200)

15 child neglect.ti,ab. (468)

16 (child$ adj2 trauma).ti,ab. (4689)

17 (non accidental injur$ or non-accidental injur$ or nonaccidental injury).ti,ab. (533)

18 Human Rights Abuses/ (734)

19 Human Trafficking/ (177)

20 ((human or person or people) adj2 (traffick$ or exploit$)).ti,ab. (818)

21 ((forced or exploit$) adj2 labour).ti,ab. (56)

22 Organ Trafficking/ (33)

23 Slavery/ (45)

24 Torture/ (1996)

25 (slavery or enslave$ or torture$).ti,ab. (2480)

26 Sex Work/ (5675)

27 (prostitut$ or brothel$).ti,ab. (3946)

28 (sex$ adj2 (exploit$ or traffick$)).ti,ab. (561)

29 Terrorism/ (4718)

30 (terrorism or terrorist$).ti,ab. (5408)

31 political terror$.ti,ab. (16)

32 Torture/ (1996)

33 exp warfare/ (35511)

34 exp Armed Conflicts/ (8467)

35 War crimes/ (1197)

36 Genocide/ (84)

37 Holocaust/ (782)

38 Ethnic Cleansing/ (0)

39 (civil adj (unrest or conflict$ or disturbance$ or war or wars or warfare)).ti,ab. (1859)

40 (persecution or victimization or victimisation).ti,ab. (7276)

41 (captivity or imprison$ or concentration camp$).ti,ab. (6151)

42 exp Disasters/ (67463)

43 Earthquakes/ (3095)

44 Tsunamis/ (708)

45 natural disaster$.ti,ab. (2928)

46 (earthquake$ or tsunami$).ti,ab. (7947)

47 (humanitarian adj (crisis or crises)).ti,ab. (221)

48 (catastrophe$ or catastrophic event$ or catastrophic experience$).ti,ab. (5922)

49 exp Survivors/ (22782)

50 Refugees/ (8204)

51 (asylum seeker$ or refugee$ or migrant$).ti,ab. (21535)

52 ((forcibly or internally) adj2 displace$).ti,ab. (476)

53 (displace$ adj2 (people or person$ or civilian$)).ti,ab. (763)

54 Crime Victims/ (7200)

55 Adult Survivors of Child Abuse/ (1437)

56 Disaster Victims/ (96)

57 Prisoners/ (15110)

58 Prisoners of War/ (469)

59 Slaves/ (31)

60 Veterans/ (13136)

61 Military Personnel/ (35416)

62 ((expose$ or exposure) adj2 (abuse or assault$ or disaster$ or terror$ or torture$ or trauma$ or rape or violen$ or war or warfare)).ti,ab. (7019)

63 (survivor$ adj2 (abuse or assault$ or disaster$ or terror$ or torture$ or trauma$ or rape or violen$ or war or warfare)).ti,ab. (2319)

64 (victim$ adj2 (abuse or assault$ or crime or disaster$ or rape or terror$ or torture$ or trauma$ or violen$ or war or warfare)).ti,ab. (8160)

65 (witness$ adj2 (abuse or assault$ or disaster$ or rape or terror$ or torture$ or trauma$ or violen$ or war or warfare)).ti,ab. (1041)

66 1 or 2 or 3 or 4 or 5 or 6 or 7 or 8 or 9 or 10 or 11 or 12 or 13 or 14 or 15 or 16 or 17 or 18 or 19 or 20 or 21 or 22 or 23 or 24 or 25 or 26 or 27 or 28 or 29 or 30 or 31 or 32 or 33 or 34 or 35 or 36 or 37 or 38 or 39 or 40 or 41 or 42 or 43 or 44 or 45 or 46 or 47 or 48 or 49 or 50 or 51 or 52 or 53 or 54 or 55 or 56 or 57 or 58 or 59 or 60 or 61 or 62 or 63 or 64 or 65 (331505)

67 Stress Disorders, Post Traumatic/ (26733)

68 (PTSD or CPTSD).ti,ab. (18107)

69 posttrauma$.ti,ab. (30066)

70 post-trauma$.ti,ab. (25637)

71 "post trauma$".ti,ab. (25637)

72 post traumatic stress.ti,ab. (9289)

73 post traumatic stress.kw. (88)

74 combat stress$.ti,ab. (343)

75 combat disorder$.ti,ab. (15)

76 DESNOS.ti,ab. (31)

77 "Disorders of Extreme Distress Not Otherwise Specified".ti,ab. (0)

78 complex trauma$.ti,ab. (403)

79 (complex adj3 trauma$).ti,ab. (1217)

80 traumatic stress.ti,ab. (10740)

81 traumatic memor$.ti,ab. (579)

82 traumatization.ti,ab. (990)

83 traumatisation.ti,ab. (192)

84 (trauma$ adj3 (expos$ or event$ or experienc$)).ti,ab. (16288)

85 67 or 68 or 69 or 70 or 71 or 72 or 73 or 74 or 75 or 76 or 77 or 78 or 79 or 80 or 81 or 82 or 83 or 84 (75076)

86 66 and 85 (24059)

87 exp Cognitive Therapy/ (22441)

88 cognitive behaviour$ therapy.ti,ab. (3982)

89 cognitive behavior$ therapy.ti,ab. (8052)

90 cognitive restructuring.ti,ab. (723)

91 cognitive rescripting.ti,ab. (0)

92 cognitive processing therapy.ti,ab. (178)

93 CPT.ti,ab. (10770)

94 cognitive therapy.ti,ab. (2397)

95 cognitive behavioural treatment$.ti,ab. (403)

96 cognitive behavioral treatment$.ti,ab. (1378)

97 (CBT or TFCBT).ti,ab. (7996)

98 cognitive trauma therapy.ti,ab. (6)

99 trauma focus$ CBT.ti,ab. (31)

100 87 or 88 or 89 or 90 or 91 or 92 or 93 or 94 or 95 or 96 or 97 or 98 or 99 (40659)

101 exp Behavior Therapy/ (64582)

102 (behavior$ adj2 (therap$ or treat$ or modif$)).ti,ab. (26789)

103 (behaviour$ adj2 (therap$ or treat$ or modif$)).ti,ab. (9862)

104 (dialectical behavio$ adj (therap$ or treat$)).ti,ab. (581)

105 biofeedback, psychology/ or feedback, sensory/ or neurofeedback/ (9330)

106 (biofeedback or neurofeedback or sensory feedback).ti,ab. (8461)

107 eye movement desensitization reprocessing/ or implosive therapy/ or virtual reality exposure therapy/ (1347)

108 (psychological adj2 desensiti$).ti,ab. (4)

109 eye movement desensiti?ation reprocessing.ti,ab. (25)

110 EMDR.ti,ab. (372)

111 (exposure adj (therap$ or treat$)).ti,ab. (2282)

112 live exposure.ti,ab. (11)

113 imaginal exposure.ti,ab. (147)

114 prolonged exposure therapy.ti,ab. (99)

115 imaginal flooding.ti,ab. (13)

116 exposure inhibition therap$.ti,ab. (0)

117 implosive therap$.ti,ab. (43)

118 image habituation.ti,ab. (3)

119 inoculation training.ti,ab. (78)

120 101 or 102 or 103 or 104 or 105 or 106 or 107 or 108 or 109 or 110 or 111 or 112 or 113 or 114 or 115 or 116 or 117 or 118 or 119 (90057)

121 "Acceptance and Commitment Therapy"/ (184)

122 (acceptance adj2 therap$).ti,ab. (257)

123 (commitment adj2 therap$).ti,ab. (603)

124 Hypnosis/ (8730)

125 (hypnosis or hypnotherap$).ti,ab. (7561)

126 Mindfulness/ (1325)

127 mindfulness.ti,ab. (4005)

128 supportive therap$.ti,ab. (3906)

129 (non-directive adj (counselling or counseling)).ti,ab. (100)

130 (nondirective adj (counselling or counseling)).ti,ab. (54)

131 (non directive adj (counselling or counseling)).ti,ab. (100)

132 Psychotherapy/ or Psychotherapy, Brief/ or Psychotherapy, Group/ or Psychotherapy, Multiple/ or Psychotherapy, Psychodynamic/ or Psychotherapy, Rational-emotive/ (65822)

133 psychodynamic therap$.ti,ab. (431)

134 inter personal psychotherap$.ti,ab. (1)

135 interpersonal psychotherap$.ti,ab. (778)

136 IPT.ti,ab. (1907)

137 (compassion adj2 therap$).ti,ab. (39)

138 accelerated resolution.ti,ab. (86)

139 sensorimotor therap$.ti,ab. (21)

140 schema therapy.ti,ab. (106)

141 (stress adj2 manag$).ti,ab. (5168)

142 supportive therap$.ti,ab. (3906)

143 Counseling/ (32854)

144 (non-directive counsel$ or non directive counsel$ or nondirective counsel$).ti,ab. (155)

145 compassion therap$.ti,ab. (1)

146 121 or 122 or 123 or 124 or 125 or 126 or 127 or 128 or 129 or 130 or 131 or 132 or 133 or 134 or 135 or 136 or 137 or 138 or 139 or 140 or 141 or 142 or 143 or 144 or 145 (122017)

147 100 or 120 or 146 (211031)

148 "hypnotics and sedatives"/ or alprazolam/ or amobarbital/ or azaperone/ or barbital/ or bromisovalum/ or chloral hydrate/ or chloralose/ or chlordiazepoxide/ or chlormethiazole/ or dexmedetomidine/ or diazepam/ or diphenhydramine/ or eszopiclone/ or ethchlorvynol/ or etomidate/ or etorphine/ or flurazepam/ or glutethimide/ or hexobarbital/ or lorazepam/ or medazepam/ or medetomidine/ or mephobarbital/ or meprobamate/ or methapyrilene/ or methaqualone/ or midazolam/ or nitrazepam/ or oxazepam/ or paraldehyde/ or pentobarbital/ or phenobarbital/ or propofol/ or secobarbital/ or temazepam/ or thiamylal/ or thiopental/ or xylazine/ (114648)

149 z drugs.ti,ab. (120)

150 anti-anxiety agents/ or bromazepam/ or buspirone/ or chlormezanone/ or clorazepate dipotassium/ or estazolam/ or flunitrazepam/ or fluvoxamine/ or nordazepam/ or ondansetron/ or oxprenolol/ or prazepam/ or pregabalin/ or ritanserin/ or tranylcypromine/ or trazodone/ or triazolam/ or zolazepam/ (33101)

151 benzodiazepines/ or benzodiazepinones/ (23660)

152 sedative antihistamine$.ti,ab. (61)

153 promethazine.ti,ab. (2061)

154 antidepressive agents/ or benactyzine/ or clorgyline/ or deanol/ or desvenlafaxine succinate/ or duloxetine hydrochloride/ or iproniazid/ or isocarboxazid/ or lithium carbonate/ or lithium compounds/ or moclobemide/ or nialamide/ or phenelzine/ or pizotyline/ or rolipram/ or sertraline/ or tranylcypromine/ or vilazodone hydrochloride/ (55533)

155 Imipramine/ (9832)

156 mirtazapine.ti,ab. (1765)

157 antipsychotic agents/ or acepromazine/ or aripiprazole/ or azaperone/ or benperidol/ or butaclamol/ or chlorpromazine/ or chlorprothixene/ or clopenthixol/ or clozapine/ or droperidol/ or etazolate/ or flupenthixol/ or fluphenazine/ or fluspirilene/ or haloperidol/ or loxapine/ or lurasidone hydrochloride/ or mesoridazine/ or methiothepin/ or methotrimeprazine/ or molindone/ or ondansetron/ or paliperidone palmitate/ or penfluridol/ or perazine/ or perphenazine/ or pimozide/ or prochlorperazine/ or promazine/ or quetiapine fumarate/ or raclopride/ or remoxipride/ or reserpine/ or risperidone/ or ritanserin/ or spiperone/ or sulpiride/ or thioridazine/ or thiothixene/ or tiapride hydrochloride/ or trifluoperazine/ or trifluperidol/ or triflupromazine/ (114997)

158 olanzapine.ti,ab. (7555)

159 anticonvulsants/ or acetazolamide/ or bromides/ or carbamazepine/ or clonazepam/ or clorazepate dipotassium/ or diazepam/ or dimethadione/ or estazolam/ or ethosuximide/ or flunarizine/ or lorazepam/ or magnesium sulfate/ or medazepam/ or mephenytoin/ or mephobarbital/ or meprobamate/ or nitrazepam/ or paraldehyde/ or phenobarbital/ or phenytoin/ or pregabalin/ or primidone/ or riluzole/ or thiopental/ or tiletamine/ or trimethadione/ or valproic acid/ or vigabatrin/ (129932)

160 antimanic agents/ or lithium chloride/ or lithium compounds/ (9229)

161 lamotrigine.ti,ab. (4632)

162 topiramate.ti,ab. (4045)

163 monoamine oxidase inhibitors/ or chlorphenamidine/ or clorgyline/ or cuprizone/ or furazolidone/ or harmaline/ or harmine/ or isocarboxazid/ or moclobemide/ or monocrotophos/ or pargyline/ or selegiline/ or tranylcypromine/ (17953)

164 Prazosin/ (7567)

165 N-Methyl-3,4-methylenedioxyamphetamine/ (3644)

166 (MDMA or ecstasy).ti,ab. (5232)

167 148 or 149 or 150 or 151 or 152 or 153 or 154 or 155 or 156 or 157 or 158 or 159 or 160 or 161 or 162 or 163 or 164 or 165 or 166 (407459)

168 86 and 147 (2717)

169 86 and 167 (405)

170 168 or 169 (3004)

171 limit 170 to yr="1992 -Current" (2818)
