# Supplementary material for: Psychological and pharmacological interventions for posttraumatic stress disorder and comorbid mental health problems following complex traumatic events: Systematic review and component network meta-analysis
Source: PLoS Med. 2020 Aug 19;17(8):e1003262. doi: 10.1371/journal.pmed.1003262 (PMC7446790; doi:10.1371/journal.pmed.1003262)
Supplement: S1 Table — ACT, acceptance and commitment therapy; CBT, cognitive behavioural therapy; DBT, dialectical behavioural therapy; EMDR, eye movement desensitisation and reprocessing; IPT, interpersonal therapy; NTCBT, non-trauma-focused CBT; MBCT, mindfulness-based cognitive therapy; MBSR, mindfulness-based stress reduction; PE, prolonged exposure; NR, not reported; RCT, randomised controlled trial; SSRI, selective serotonin reuptake inhibitor; STAIR, skills training in affective and interpersonal regulation; TAU, treatment as usual; TFCBT, trauma-focused CBT. (DOCX) [file pmed.1003262.s003.docx]

S1 Table Characteristics of included studies

| **Study** | **Country** | **Trauma exposure category** | **Intervention(s)** | **Intervention category** | **Control(s)** | **Intensity (duration, frequency and length of sessions)** | **Study design** | **Risk of bias (overall)** |
| --- | --- | --- | --- | --- | --- | --- | --- | --- |
| Acarturk et al[1] | Turkey/Syria | Refugee | EMDR | EMDR | Waitlist | Duration: 7 sessions Frequency: NR Length of sessions: 90 minutes | RCT | Low |
|  |  |  |  |  |  |  |  |  |
|  |  |  |  |  |  |  |  |  |
| Acarturk et al[2] | Turkey/Syria | Refugee | EMDR | EMDR | Waitlist | Duration: 5 weeks Frequency: NR Length of sessions: NR | RCT | Low |
|  |  |  |  |  |  |  |  |  |
|  |  |  |  |  |  |  |  |  |
| Adenauer et al[3] | Germany | Refugee | Narrative exposure therapy | TFCBT | Waitlist | Duration: 12 sessions Frequency: Weekly or biweekly Length of sessions: average 108 minutes | RCT | Moderate |
|  |  |  |  |  |  |  |  |  |
|  |  |  |  |  |  |  |  |  |
| Ardani et al[4] | Iran | Veterans | Rivastigmine augmented therapy | Rivastigmine | Placebo | Duration: 12 weeks Frequency: twice a day Dose: Week 1-4: 1.5mg 2x a day, Week 5-:3mg 2x a day in addition to citalopram 40mg/day and sodium valproate 20mg/kg /day. | RCT | Low |
| Azad et al[5] | Iran | War-related | Mindfulness-based stress reduction | Mindfulness | Waitlist | Duration: 8 sessions, 4 weeks Frequency: Twice a week Length of sessions: 90 minutes | RCT | High |
| Bahadir-Yilmaz et al[6] | Turkey | Domestic violence | Empowerment program | TFCBT | Control | Duration: 10 weeks Frequency: Weekly Length of sessions: 90 minutes | RCT | High |
| Bartozkis et al[7] | USA | Veterans | Risperidone | Antipsychotic | Placebo | Duration: 16 weeks Frequency: NR Dose: Initiated at 1mg, increased to 3mg bedtime dose | RCT | High |
| Bass et al[8] | Iraq | War-related | Trauma-informed counselling | TFCBT | Control | Duration: 6-12 sessions Frequency: NR Length of sessions: NR | RCT | Low |
| Becker et al[9] | USA | Veterans | Bupropion SR | Antidepressant | Placebo | Duration: 8 weeks Frequency: Once-twice a day.  Dose: 100mg/day for 2 weeks. Dose increased to 100mg/twice a day as indicated. | RCT | Moderate |
| Beidel et al[10] | USA | Veterans | Trauma Management Therapy with Exposure | TFCBT | Exposure only | Duration: 17 weeks Frequency: Exposure 3x/week; social and emotional rehabilitation twice a week for 10 weeks. Length of sessions: 190 minutes | RCT | High |
|  |  |  |  |  |  |  |  |  |
| Bichescu et al[11] | Romania | War-related | Narrative exposure therapy | TFCBT | Psychoeducation | Duration: 5 weeks Frequency: weekly or biweekly  Length of sessions: 120 minutes | RCT | High |
| Bolton et al[12] | Iraqi-Kurdistan | War-related | Behavioural Activation Treatment for Depression | NTFCBT | Waitlist | Duration: 12 sessions Frequency: NR Length of sessions: NR | RCT | Low |
|  |  |  | Cognitive Processing Therapy | TFCBT |  | Duration: 12 sessions Frequency: NR Length of sessions: NR |  |  |
| Bonillar-Escobar et al[13] | Colombia | War-related | Common Elements Treatment Approach | TFCBT | Waitlist | Duration:12-14 weeks Frequency: weekly Length of sessions: 90 minutes | RCT | Moderate |
| Bremner et al[14] | USA | Veterans | Mindfulness-based stress reduction | Mindfulness | Active control | Duration: 8 weeks Frequency: weekly Length of sessions: 2.5 hours (+ one all day) | RCT | High |
| Buhmann et al[15] | Denmark | Refugee | Sertraline, psychoeducation and CBT | TFCBT/Antidepressant | Waitlist | Duration: 16 sessions, 6 months Frequency: psychoeducation & CBT weekly/sertraline daily Dose/length of sessions: Sertraline increased 25-50mg to a maximum dose of 200mg. Mianserin given at 10-30mg, titrated weekly by 10mg | RCT | Low |
|  |  |  | Sertraline and psychoeducation | SSRI/Antidperessant/other psyshotherapy |  | Duration: 10 sessions, 6 months Frequency: psychoeducation weekly/sertraline daily Dose/length of sessions: NR. |  |  |
| Carlson et al[16] | USA | Veterans | EMDR Biofeedback relaxation | EMDR | Waitlist | Duration: 12 sessions, 6 weeks  Frequency: twice a week  Length of sessions: 60-75 minutes | RCT | High |
|  |  |  | Biofeedback relaxation | NTFCBT |  | Duration: 12 sessions, 6 weeks  Frequency: twice a week  Length of sessions: 40 minutes |  |  |
| Carlson et al[17] | Denmark | Refugee | Stress management | NTFCBT |  | Duration: 26 sessions, 6-7 months Frequency: NR Length of sessions:45-60 minutes | RCT | Low |
|  |  |  | Cognitive restructuring | NTFCBT |  |  |  |  |
| Celik et al[18] | Turkey | Veterans | Paraxetine | SSRI |  | Duration: 8 weeks Frequency: once a day Dose: Initiated at 10mg/day for week 1, then increased to 20mg for weeks 2 and 3, 30mg for 4 and 5, 40mg for 6 and 7, and 60mg weeks 8-12 (if tolerated/clinically indicated | RCT | High |
|  |  |  | Amitriptyline | Antidepressant |  | Duration: 8 weeks Frequency: once a day Dose: Initiated at 75mg/day for weeks 1-3 (initial dose of 25mg for days 1-3, 50mg, 4-7, and 75mg for days 8-21), then increased to 100mg from week 4 (as necessary), and up to 200mg/down to 75mg from week 5 (as necessary). |  |  |
| Chard et al[19] | USA | Childhood sexual abuse | Cognitive Processing Therapy – for Sexual Abuse survivors | TFCBT | Waitlist | Duration: 17 weeks Frequency: weekly individual and group sessions  Length of sessions: group sessions 90 minutes; individual sessions 60 minutes | RCT | Moderate |
| Chung et al[20] | South Korea | Veterans | Mirtazapine | Antidepressant |  | Duration: 6 weeks Frequency: daily Dose: initiated at mean dose 19.1± 8.53mg/day, weeks 1–2 31.5± 9.13mg/day, weeks 2–6 38.5± 12.10 mg/day. | RCT | High |
|  |  |  | Sertaline | SSRI |  | Duration: 6 weeks Frequency: daily Dose: initiated at mean dose 19.1± 8.53mg/day, weeks 1–2 31.5± 9.13mg/day, weeks 2–6 38.5± 12.10 mg/day. |  |  |
| Classen et al[21] | USA | Childhood sexual abuse | Trauma-focused therapy | TFCBT | Waitlist | Duration: 24 weeks Frequency: weekly Length of sessions: 90 minutes | RCT | Low |
|  |  |  | Present centred therapy |  |  | Duration: 24 weeks Frequency: weekly Length of sessions: 90 minutes |  |  |
| Cloitre et al[22] | USA | Childhood sexual abuse | STAIR-modified PE | DBT | Minimal attention waitlist | Duration: 12 weeks, 16 sessions Frequency: Weekly STAIR/twice weekly prolonged exposure  Length of sessions: STAIR 60 minutes, modified prolonged exposure 90 minutes. | RCT | Moderate |
|  |  |  | STAIR/Exposure | DBT |  | Duration: 16 weeks Frequency: weekly Length of sessions: NR |  |  |
|  |  |  | STAIR/Support | DBT |  | Duration: 16 weeks Frequency: weekly Length of sessions: NR |  |  |
| Cook et al[23] | USA | Veterans | Imagery Rehearsal therapy | TFCBT |  | Duration: 6 weeks Frequency: weekly Length of sessions: 90 minutes | RCT | Low |
|  |  |  | Sleep and nightmare management |  |  | Duration: 6 weeks Frequency: weekly Length of sessions: 90 minutes |  |  |
| Davis et al[24] | USA | Veterans | Nefazodone | Antidepressant | Placebo | Duration: 12 weeks Frequency: twice a day Dose: Initiated at 100mg, increased 100mg every 4 days as tolerated, up to a maximum benefit (not exceeding 600mg/day). | RCT | Low |
| Davis et al[25] | USA | Veterans | Divalproex | Anticonvulsant | Placebo | Duration: 12 weeks Frequency: twice a day Dose: Initiated at 100mg, increased 100mg every 4 days as tolerated, up to a maximum benefit (not exceeding 600mg/day). | RCT | Low |
| Devilly et al[26] | USA | Veterans | EMDR | EMDR | Standard psychiatric support | Duration: 2 sessions Frequency: NR Length of sessions: 90 minutes | RCT | Moderate |
|  |  |  | Reactive Eye Dilation Desensitization and Reprocessing | EMDR |  | Duration: 2 sessions Frequency: NR Length of sessions: 90 minutes |  |  |
| Edmond et al[27] | USA | Childhood sexual abuse | EMDR | EMDR | Delayed treatment | Duration: 6 weeks Frequency: weekly Length of sessions: 90 minutes | RCT | Moderate |
|  |  |  | Routine individual treatment |  |  | Duration: 6 weeks Frequency: weekly Length of sessions: 90 minutes |  |  |
| Engel et al[28] | USA | Veterans | DESTRESS-PC | NTFCBT | Optimised usual care | Duration: 18 sessions, 6 weeks Frequency: 3x/week  Length of sessions: 15-30 minutes, homework 30 minutes | RCT | Low |
| Feske et al[29] | USA | Mixed | Prolonged exposure | TFCBT | Treatment as usual | Duration: 9-12 sessions, 12 weeks Frequency: weekly Length of sessions: 90 minutes | RCT | Moderate |
| Foa et al[30] | USA | Veterans | Massed prolonged exposure therapy | TFCBT |  | Duration: 2 weeks Frequency: daily Length of sessions: NR | RCT | Low |
|  |  |  | Spaced prolonged exposure therapy | TFCBT |  | Duration: 8 weeks Frequency: once or twice weekly Length of sessions: NR |  |  |
|  |  |  | Present centred therapy | NTFCBT |  | Duration: 10 sessions Frequency: once or twice weekly Length of sessions: 90 minutes, |  |  |
| Ford et al[31] | USA | Veterans | Prolonged exposure | TFCBT | Trauma Affect Regulation: Guide for Education and Therapy | Duration: 10 weeks Frequency: Weekly Length of sessions: 75-90 minutes | RCT | High |
| Franciskovic et al[32] | Croatia | Mixed | Tianeptine and group therapy | Antidepressant and other psychotherapy |  | Duration: 6 months Frequency: group therapy twice weekly/tianeptine daily Dose/length of sessions: 37.5mg/day. NR for group therapy. | RCT | Moderate |
|  |  |  | Fluoxetine and group therapy | SSRI and other psychotherapy |  | Duration: 6 months Frequency: group therapy twice weekly/fluoxetine daily Dose/length of sessions: 40mg/day. NR for group therapy. |  |  |
| Franklin et al[33] | USA | Veterans | Prolonged exposure by iPhone | TFCBT | Treatment as usual | Duration: 10 sessions, 10 weeks  Frequency: NR  Length of sessions: NR | RCT | Low |
|  |  |  | Prolonged exposure by computer-based teleconferencing | TFCBT | Treatment as usual | Duration: 10 sessions, 10 weeks  Frequency: NR Length of sessions: NR |  |  |
| Friedman et al[34] | USA | Veterans | Sertraline | SSRI | Placebo | Duration: 12 weeks Frequency: Daily Dose: Initiated at 25mg/day, week 2 increased dose to 50mg/day where dose-limiting adverse events did not present. | RCT | Low |
| Galano et al[35] | USA | Domestic violence | Women’s empowerment program | TFCBT | Control | Duration: 10 weeks Frequency: weekly Length of sessions: NR | RCT | Moderate |
| Gamito et al[36] | Portugal | Veterans | Virtual reality exposure therapy | TFCBT | Waitlist | Duration: 12 sessions Frequency: NR Length of sessions: NR | RCT | Moderate |
|  |  |  | Exposure in imagination | TFCBT |  | Duration: 12 sessions Frequency: NR Length of sessions: NR |  |  |
| Germain et al[37] | Portugal | Veterans | Prazosin | Prazosin | Placebo | Duration: 8 weeks Frequency: daily Dose: Mean dose 8.9mg. | RCT | Moderate |
| Hamner et al[38] | USA | Veterans | Adjunctive risperidone | Antipsychotic | Placebo | Duration: 5 weeks Frequency: Daily Dose: Initiated at 1mg/day, increased to a maximum of 6mg/day. | RCT | Low |
| Hermenau et al[39] | Democratic Republic of Congo | War-related | Narrative Exposure Therapy for Forensic Offender Rehabilitation (FORNET) | TFCBT | No intervention | Duration: 2 weeks Frequency: approximately every other day Length of sessions: 1-2 hours | RCT | Low |
| Hijazi et al[40] | USA | Refugee | Brief Narrative Exposure therapy | TFCBT | Waitlist | Duration: 3 weeks Frequency: weekly Length of sessions: 60-90 minutes | RCT | Low |
| Himmerich et al[41] | Germany | Veterans | Inpatient psychotherapy | EMDR | Outpatient clinical management | Duration: 6 weeks Frequency: NR Length of sessions: NR | RCT | High |
| Hinton et al[42] | USA | Refugee | Cognitive Behavioural Therapy | TFCBT | Delayed treatment | Duration: 11 weeks Frequency: weekly Length of sessions: NR | RCT | Moderate |
| Hinton et al[43] | USA | Refugee | Cognitive Behaviour Therapy | TFCBT | Delayed treatment | Duration: 12 weeks Frequency: weekly Length of sessions: NR | RCT | High |
| Jensen et al[44] | USA | Veterans | EMDR | EMDR | Treatment as usual with option of delayed EMDR | Duration: NR Frequency: NR Length of sessions: NR | RCT | High |
| Jung et al[45] | Germany | Childhood sexual abuse | Cognitive restructuring & Imagery Modification | TFCBT | Waitlist | Duration: 2 sessions Frequency: NR Length of sessions: treatment 90 minutes, booster 50 minutes | RCT | High |
| Katz et al[46] | USA | Mixed | Holographic reprocessing | Other psychotherapy | Person-centred control | Duration: 10 weeks Frequency: weekly Length of sessions: NR | RCT | High |
|  |  |  | Prolonged exposure | TFCBT | Waitlist |  |  |  |
| Keane et al[47] | USA | Veterans | Implosive (Flooding) Therapy | TFCBT | Waitlist | Duration: 14 sessions Frequency: NR Length of sessions: 90 minutes | RCT | High |
| Kearney et al[48] | USA | Veterans | MBSR | Mindfulness | TAU | Duration: 8 weeks Frequency: weekly Length of sessions: 2.5 hours (+7 hour retreat) | RCT | Low |
| King et al[49] | USA | Veterans | MBCT | Mindfulness | TAU | Duration: 8 weeks Frequency: weekly Length of sessions: 8 hours | Non-RCT | Moderate |
| Knaevelsrud et al[50] | Iran/Germany | War-related | Internet-based CBT | TFCBT | Waitlist | Duration: 5 weeks Frequency: twice a week  Length of sessions: not fixed | RCT | Low |
| Knaevelsrud et al[51] | Germany | War-related | Web-based testimonial therapy | TFCBT | Control | Duration: 6 weeks Frequency: twice a week Length of sessions: 45 minutes | RCT | Low |
| Kosten et al[52] | USA | Veterans | Phenelzine and psychotherapy | Antidepressant and TFCBT | Placebo and psychotherapy | Duration: NR Frequency: psychotherapy weekly/imipramine daily Dose/length of sessions: dose of drug and length of sessions NR. Mean maximal dose 68+/- 20mg. | RCT | Moderate |
|  |  |  | Imipramine and psychotherapy | Antidepressant and TFCBT |  | Duration: NR Frequency: psychotherapy weekly/imipramine daily Dose/length of sessions: dose of drug and length of sessions NR. Mean maximal dose 225+/- 55mg. |  |  |
| Krupnick et al[53] | USA | Childhood sexual abuse | Interpersonal psychotherapy | IPT | Waitlist | Duration: 16 weeks Frequency: weekly Length of sessions: 2 hours | RCT | Moderate |
| Kruse et al[54] | USA | Veterans | Trauma-focused psychotherapy | TFCBT | TAU | Duration: NR Frequency: weekly (3 months), fortnightly (remainder) Length of sessions: 50 mins, 25 hours total. | Non-RCT | Low |
| Krystal et al[55] | USA | Veterans | Adjunctive risperidone | Antipsychotic | Placebo | Duration: 24 weeks Frequency: daily Dose: Initiated at 1mg at night. Increased 1mg/week to 3mg. | RCT | Low |
| Kubany et al[56] | USA | Domestic violence | Cognitive Trauma Therapy for Battered Women | TFCBT | Waitlist | Duration: 4 weeks Frequency: NR Length of sessions: 60 minutes. | RCT | High |
| Kubany et al[57] | USA | Domestic violence | Cognitive Trauma Therapy for Battered Women | TFCBT | Delayed treatment | Duration: 8-11 sessions, 4-6 weeks Frequency: twice a week  Length of sessions: 60 minutes | RCT | High |
| Lande et al[58] | USA | Veterans | Biofeeedback | Biofeedback | TAU | Duration: 3 weeks Frequency: twice a week  Length of sessions: 20 minutes | RCT | High |
| Lau et al[59] | Denmark | Childhood sexual abuse | Analytic group psychotherapy | Other psychotherapy | TAU | Duration: 52 weeks Frequency: weekly Length of sessions: 2.25 hours | RCT | Low |
|  |  |  | Systemic group psychotherapy | Other psychotherapy |  |  |  |  |
| Levi et al[60] | Israel | Veterans | CBT |  |  | Duration: 24 weeks Frequency: weekly Length of sessions: NR | Non-RCT | High |
|  |  |  | Psychodynamic therapy |  |  | Duration: 50 weeks Frequency: weekly Dose/length of sessions: NR |  |  |
| Lindley et al[61] | USA | Veterans | Topiramate | Anticonvulsant | Placebo | Duration: 7 weeks Frequency: daily Dose: Initiated at 25mg, increased 50mg each week until maximum toleration or 200mg/day. | RCT | High |
| Lundqvist et al[62] | Sweden | Childhood sexual abuse | Group therapy |  | Waitlist | Duration: 2 years (Phase 1: 5 months, Phase 2: 4 months, Phase 3: 1 year). Frequency: Phase 1: twice a week, Phase 2: weekly, Phase 3: monthly Length of sessions: NR | Non-RCT | Moderate |
|  |  |  | Short-term focused therapy |  |  | Duration: 2 years (Phase 1: 5 months, Phase 2: 4 months, Phase 3: 1 year). Frequency: Phase 1: twice a week, Phase 2: weekly, Phase 3: monthly Length of sessions: NR |  |  |
| Margolies et al[63] | USA | Veterans | Cognitive Behavioural Therapy for Insomnia with Imagery Rehearsal Therapy | TFCBT | Minimal attention wait-list | Duration: 8 weeks Frequency: NR Length of sessions: 60 minutes. | RCT | Moderate |
| McDonagh et al[64] | USA | Childhood sexual abuse | Cognitive Behavioural Therapy | TFCBT | Minimal attention wait-list | Duration: 14 weeks Frequency: weekly Length of sessions: 2 hours first 7 sessions; 1.5 hours final 7 sessions | RCT | Moderate |
|  |  |  | Present centred therapy | Other psychotherapy |  | Duration: 14 weeks Frequency: weekly Length of sessions: 2 hours first 7 sessions; 1.5 hours final 7 sessions |  |  |
| McLay et al[65] | USA | Veterans | Virtual Reality Graded Exposure Therapy | TFCBT | Waitlist | Duration: 10 weeks Frequency: up to twice a week  Length of sessions: NR | RCT | High |
| Meffert et al[66] | Egypt | Refugee | Interpersonal Psychotherapy | IPT | Waitlist | Duration: 3 weeks Frequency: twice a week Length of sessions: NR | RCT | High |
| Miyahira et al[67] | USA | War-related | Add-on Virtual Reality Exposure | TFCBT | Minimal attention | Duration: 5 weeks Frequency: twice a week  Length of sessions: NR | RCT | High |
| Monnelly et al[68] | USA | Veterans | Risperidone | Antipsychotic | Placebo | Duration: 6 weeks Frequency: daily Dose: Initiated at 0.5mg, increased fortnightly to maximum 2mg/day. | RCT | High |
| Moradi et al[69] | Iran | Veterans | Memory Specificity Training | NTFCBT | Treatment as usual | Duration: 4 sessions, 4 weeks  Frequency: weekly  Length of sessions: 1 hour | RCT | Moderate |
| Morgan et al[70] | Canada | Childhood sexual abuse | EMDR |  | Waitlist | Duration: 70 days (typically) Frequency: Dose/length of sessions: 90 minutes | Non-RCT | Moderate |
|  |  |  | CBT |  |  | Duration: 70 days Frequency: weekly Length of sessions: 60-120 minutes |  |  |
| Nakamura et al[71] | USA | Veterans | Mind-body bridging sleep intervention | Mindfulness | Active control | Duration: 3 weeks Frequency: weekly Length of sessions: NR | RCT | Low |
| Narimani et al[72] | Iran | War-related | EMDR |  | Waitlist | Duration: 70 days (typically) Frequency: NR Dose/length of sessions: 90 minutes | Non-RCT | High |
|  |  |  | CBT |  |  | Duration: 70 days Frequency: NR Length of sessions: 60-120 minutes |  |  |
| Naylor et al[73] | USA | Veterans | Paroxetine | SSRI | Placebo | Duration: 12 weeks Frequency: daily Dose: flexible dosing 10-40mg/day depending on tolerance and response. | RCT | High |
| Neuner et al[74] | Uganda | Refugee | Narrative exposure therapy | TFCBT | Monitoring | Duration: 3 weeks Frequency: twice a week  Length of sessions: 2 hours | RCT | Low |
|  |  |  | Trauma counselling | TFCBT |  | Duration: 3 weeks Frequency: twice a week  Length of sessions: 2 hours |  |  |
| Neuner et al[75] | Uganda | Refugee | Narrative exposure therapy | TFCBT | Psychoeducation | Duration: 4 sessions, 3 weeks Frequency: 4 sessions in 3 weeks Length of sessions: 90 minutes | RCT | High |
|  |  |  | Supportive counselling | TFCBT |  | Duration: 4 sessions, 3 weeks Frequency: 4 sessions in 3 weeks Length of sessions: 90 minutes |  |  |
| Niles et al[76] | USA | Veterans | Mindfulness | Mindfulness | Psychoeducation | Duration: 8 weeks Frequency: weekly Length of sessions: 45 minutes in-person sessions, 20 minutes telephone sessions. | RCT | High |
| Otto et al[77] | USA | Refugee | CBT and Sertraline | TFCBT and SSRI |  | Duration: NR Frequency: CBT NR/ sertraline daily Dose/length of sessions: CBT 10 sessions, length NR.  Sertraline initiated at 25mg, titrated 50mg each week to maximum 200mg. Mean final dose 100mg/d. | RCT | High |
| Owens et al[78] | USA | Childhood sexual abuse | Cognitive Processing Therapy for Sexual Abuse | TFCBT | Minimal attention | Duration: 17 weeks Frequency: NT Length of sessions: NR | RCT | Moderate |
| Panahi et al[79] | Iran | Veterans | Sertraline | SSRI | Placebo | Duration: 10 weeks Frequency: daily Dose: initiated at 50mg/day, flexible adjustment fortnightly to a maximum of 200mg/day. | RCT | Low |
| Paunovic et al[80] | Sweden | Refugee | Cognitive behavioural therapy | TFCBT | Minimal attention | Duration: 4-5 months Frequency: weekly Length of sessions: NR | RCT | High |
|  |  |  | Exposure only therapy | Exposure only |  | Duration: 4-5 months Frequency: weekly Length of sessions: 20 minutes |  |  |
| Pivac et al[81] | Croatia | Veterans | Olanzapine |  |  | Duration: 6 weeks Frequency: Once or twice daily Dose: 5-10mg | Non-RCT | Moderate |
|  |  |  | Fluphenazine |  |  | Duration: 6 weeks Frequency: Once or twice daily Dose: 5-10mg |  |  |
| Polusny et al[82] | USA | Veterans | Mindfulness-based stress reduction | Mindfulness |  | Duration: 9 weeks Frequency: weekly Length of sessions: 2.5 hours | RCT | Low |
|  |  |  | Present centred therapy | Other psychotherapy |  | Duration: 9 weeks Frequency: weekly Length of sessions: 1.5 hours |  |  |
| Possemato et al[83] | USA | Veterans | Primary care brief mindfulness training | Mindfulness | TAU | Duration: 4 weeks Frequency: weekly Length of sessions: 1.5 hours | RCT | Low |
| Raskind et al[84] | USA | Veterans | Prazosin | Prazosin | Placebo | Duration: 8 weeks Frequency: daily Dose: Initiated at 1mg/nightly for 3 days. Days 3-7 increased to 2mg/day based on response. Persistence of traumatic nightmares increased dose to 2mg per week up to 10mg/day at day 28. Persistent lack of response allowed additional 5mg to a maximum of 15mg/day. | RCT | Low |
| Raskind et al[85] | USA | Veterans | Prazosin | Prazosin | Placebo | Duration: 15 weeks Frequency: Once to twice daily Dose: Titrated 1-2mg/day, depending on gender | RCT | Low |
| Rauch et al[86] | USA | Veterans | Prolonged exposure + placebo or sertraline | TFCBT; SSRI | Active control | Duration: 24 weeks Frequency: variable (13 sessions in 24 weeks) Length of sessions: 90 minutes | RCT | Low |
| Ready et al[87] | USA | Veterans | Virtual Reality Exposure | TFCBT |  | Duration: 10 sessions Frequency: NR Length of sessions: 90 minutes | RCT | Low |
|  |  |  | Present centred therapy | Other psychotherapy |  | Duration: 10 sessions Frequency: NR Length of sessions: 90 minutes |  |  |
| Reed et al[88] | USA | Domestic violence | Forgiveness Therapy | Other psychotherapy | Alternative therapy | Duration: 5-12 months Frequency: weekly Length of sessions: 1 hour | RCT | High |
| Reger et al[89] | USA | Veterans | Virtual Reality Exposure | TFCBT | Waitlist | Duration: 10 sessions, 5 weeks Frequency: weekly or twice weekly  Length of sessions: 1.5-2 hours | RCT | Low |
|  |  |  | Prolonged Exposure | TFCBT |  | Duration: 10 sessions, 5 weeks Frequency: weekly or twice weekly  Length of sessions: 1.5-2 hours |  |  |
| Reich et al[90] | USA | Childhood sexual abuse | Risperidone | Antipsychotic | Placebo | Duration: 8 weeks Frequency: daily (can be divided into 2-3 doses) Dose: initiated at 0.5mg/day, increased to 1mg/day at 3 days, then up to 1mg/day/week up to target dose of 4mg/day | RCT | High |
| Resick et al[91] | USA | Veterans | Cognitive Processing Therapy – Cognitive only version | TFCBT | Active | Duration: 12 sessions Frequency: twice a week Length of sessions: 90 minutes | RCT | Low |
|  |  |  | Present centred therapy | Other psychotherapy |  | Duration: 12 sessions Frequency: twice a week Length of sessions: 90 minutes |  |  |
| Rogers et al[92] | USA | Veterans | EMDR | EMDR | Active | Duration: 1 session Frequency: once Length of sessions: 60-90 minutes | RCT | Low |
|  |  |  | Exposure (implosive flooding) | Exposure only |  | Duration: 1 session Frequency: once Length of sessions: 60-90 minutes |  |  |
| Salo et al[93] | Palestine | War-related | Individual therapy |  | No intervention | Duration: 1 year Frequency: weekly Length of sessions: NR | Non-RCT | Low |
|  |  |  | Group therapy |  |  | Duration: 1 year Frequency: weekly Dose/length of sessions: NR |  |  |
| Saxe et al[94] | Canada | Childhood sexual abuse | Victim to Survivor’ group therapy |  | Waitlist | Duration: 20 weeks Frequency: weekly Dose/length of sessions: NR | Non-RCT | High |
| Shapiro et al[95] | Israel | War-related | EMDR | EMDR | Control | Duration: 3 days Frequency: once a day Length of sessions: 90 minutes | RCT | Low |
| Sikkema et al[96] | USA | Childhood sexual abuse | HIV and trauma coping group | NTFCBT | Waitlist | Duration: 15 weeks Frequency: weekly Length of sessions: 90 minutes | RCT | High |
|  |  |  | Support group | NTFCBT |  | Duration: 15 weeks Frequency: weekly Length of sessions: 90 minutes |  |  |
| Sikkema et al[97] | USA | Childhood sexual abuse | Coping skills programme | NTFCBT | HIV Support group | Duration: 15 weeks Frequency: weekly Length of sessions: 90 minutes | RCT | High |
| Smajkic et al[98] | USA | Refugee | Paroxetine | SSRI |  | Duration:6 weeks Frequency: daily.  Dose: 20 mg once daily for 14 days, then if tolerated at 2 weeks, the dosage was continued. | RCT | High |
|  |  |  | Sertraline | SSRi |  | Duration:6 weeks Frequency: daily.  Dose: 50 mg once daily for 14 days, then if tolerated at 2 weeks, 100 mg once daily. |  |  |
| Sonne et al[99] | Denmark | Refugees | Venlafaxine | Antidepressant | Placebo | Duration:6 weeks Frequency: twice a day  Dose: 37.5 mg twice daily for 14 days, then if tolerated at 2 weeks, 75 mg twice daily. | RCT | High |
| Spidel et al[100] | Canada | Childhood sexual abuse | ACT | Mindfulness | Control | Duration: 8 sessions Frequency: NR Length of sessions: 70-75 minutes | RCT | Moderate |
| Stein et al[101] | USA | Veterans | Adjunctive olanzapine | Antipsychotic | Placebo | Duration: 8 weeks Frequency: daily Dose:  Initiated at 10mg, increased to 20mg after 2 weeks if clinically indicated and tolerated. | RCT | Moderate |
| Stenmark et al[102] | Germany | Refugee | Narrative exposure therapy | TFCBT | Treatment as usual | Duration: 10 weeks Frequency: weekly Length of sessions: 90 minutes | RCT | High |
| Teng et al[103] | USA | Veterans | Panic Control Treatment | NTFCBT | Psychoeducation | Duration: 10 weeks Frequency: weekly Length of sessions: 1 hour | RCT | Moderate |
| Ter Heide et al[104] | The Netherlands | Refugee | EMDR | EMDR |  | Duration: 11 weeks Frequency: weekly Length of sessions: 90 minutes | RCT | High |
|  |  |  | Stabilisation | EMDR |  | Duration: 11 weeks Frequency: weekly Length of sessions: 60 minutes |  |  |
| Ter Heide et al[105] | The Netherlands | Refugee | EMDR | EMDR |  | Duration: 9 weeks Frequency: NR Length of sessions: NR | RCT | High |
|  |  |  | Stabilisation-as-usual | Stabilisation |  | Duration: 9 weeks Frequency: NR Length of sessions: NR |  |  |
| Thorp et al[106] | USA | Veterans | Prolonged exposure | TFCBT | Relaxation therapy | Duration: 12 weeks Frequency: weekly Length of sessions: 90 minutes | RCT | High |
| Ulmer et al[107] | USA | Veterans | Sleep Intervention for PTSD | TFCBT | Usual care | Duration: 12 weeks Frequency: fortnightly Length of sessions: 60 minutes | RCT | Moderate |
| Van der Kolk et al[108] | USA | Mixed | Fluoxetine | SSRI | Placebo | Duration: 5 weeks Frequency: daily Dose: Initiated at 20mg/day, increased weekly to a maximum of 60mg. | RCT | Moderate |
| Wahbeh et al[109] | USA | Veterans | Mindfulness Meditation | Mindfulness | Sitting Quietly | Duration: 6 weeks Frequency: weekly Length of sessions: 20 minutes | RCT | High |
|  |  |  | Slow Breathing with biofeedback | Biofeedback |  | Duration: 6 weeks Frequency: weekly Length of sessions: 20 minutes |  |  |
|  |  |  | Slow breathing | Other psychological intervention |  | Duration: 6 weeks Frequency: weekly Length of sessions: 20 minutes |  |  |
| Wang et al[110] | Kosovo | War-related | Biofeedback-supported CBT | TFCBT | Waitlist | Duration: 10 weeks Frequency: twice a week  Length of sessions: 60-90 minutes | RCT | Low |
| Weiss et al trial 1: CETA[111] | Iraq | War-related | Common Elements Treatment Approach | TFCBT | Waitlist | Duration: 8-12 weeks Frequency: weekly Length of sessions: 50-60 minutes | RCT | Low |
| Weiss et al trial 2: CPT[111] | Iraq | War-related | Cognitive Processing Therapy | TFCBT | Waitlist | Duration: 12 weeks Frequency: weekly Length of sessions: NR | RCT | Low |
| Yeomans et al[112] | Burundi | War-related | Workshop with psychoeducation | Other psychotherapy | Waitlist | Duration: 4 days Frequency: 3 consecutive days and a month follow-up Length of sessions: day long | RCT | High |
|  |  |  | Workshop without psychoeducation | Other psychotherapy |  | Duration: 4 days Frequency: 3 consecutive days and a month follow-up Length of sessions: day long |  |  |
| Yurtsever et al[113] | Turkey | Refugee | EMDR | EMDR |  | Duration: 3 days Frequency: 2 sessions in 3 days Length of sessions: 4 hours | RCT | Low |
| Zlotnick et al[114] | USA | Childhood sexual abuse | Affect-Management |  | Waitlist | Duration: 15 weeks Frequency: weekly Length of sessions: 180 minutes | RCT | High |
| Zohar et al[115] | Israel | Veterans | Sertraline | SSRI | Placebo | Duration: 10 weeks Frequency: daily Dose: initiated at 50mg/day, flexible titration in 50mg increments fortnightly up to a maximum 200mg. | RCT | High |

ACT – acceptance and commitment therapy, CBT – cognitive behavioural therapy, DBT – dialectical behavioural therapy, EMDR – eye movement desensitization and reprocessing, IPT – interpersonal therapy, NTCBT – non-trauma focused CBT, MBCT – mindfulness based cognitive therapy, MBSR – mindfulness based stress reduction, PE – prolonged exposure, NR – not reported, RCT – randomised controlled trial, SSRI – selective serotonin reuptake inhibitor, STAIR – skills training in affective and interpersonal regulation, TAU – treatment as usual, TFCBT – trauma-focused CBT.

**References**

1. Acarturk C, Konuk E, Cetinkaya M, Senay I, Sijbrandij M, Cuijpers P, et al. EMDR for Syrian refugees with posttraumatic stress disorder symptoms: results of a pilot randomized controlled trial. European Journal of Psychotraumatology. 2015;6:27414. doi: <https://dx.doi.org/10.3402/ejpt.v6.27414> PubMed PMID: 25989952.

2. Acarturk C, Konuk E, Cetinkaya M, Senay I, Sijbrandij M, Gulen B, et al. The efficacy of eye movement desensitization and reprocessing for post-traumatic stress disorder and depression among Syrian refugees: results of a randomized controlled trial. Psychological Medicine. 2016;46(12):2583-93. doi: <https://dx.doi.org/10.1017/S0033291716001070> PubMed PMID: 27353367.

3. Adenauer H, Catani C, Gola H, Keil J, Ruf M, Schauer M, et al. Narrative exposure therapy for PTSD increases top-down processing of aversive stimuli--evidence from a randomized controlled treatment trial. BMC Neuroscience. 2011;12:127. doi: <https://dx.doi.org/10.1186/1471-2202-12-127> PubMed PMID: 22182346.

4. Ardani AR, Hosseini G, Bordbar MR, Talaei A, Toroghi HM. Effect of rivastigmine augmentation in treatment of male patients with combat-related chronic posttraumatic stress disorder a randomized controlled trial. Journal of Clinical Psychopharmacology (USA). 2017;37(1):54-60. doi: 10.1097/JCP.0000000000000624.

5. Azad Marzabadi E, Hashemi Zadeh SM. The effectiveness of mindfulness training in improving the quality of life of the war victims with post traumatic stress disorder (PTSD). Iranian Journal of Psychiatry. 2014;9(4):228-36. PubMed PMID: 25792991.

6. Bahadir-Yilmaz E, Oz F. The Effectiveness of Empowerment Program on Increasing Self-Esteem, Learned Resourcefulness, and Coping Ways in Women Exposed to Domestic Violence. Issues in Mental Health Nursing. 2018;39(2):135-41. doi: 10.1080/01612840.2017.1368750. PubMed PMID: WOS:000424943000005.

7. Bartzokis G, Lu PH, Turner J, Mintz J, Saunders CS. Adjunctive risperidone in the treatment of chronic combat-related posttraumatic stress disorder. Biological Psychiatry. 2005;57(5):474-9. doi: 10.1016/j.biopsych.2004.11.039. PubMed PMID: 15737661.

8. Bass J, Murray SM, Mohammed TA, Bunn M, Gorman W, Ahmed AM, et al. A Randomized Controlled Trial of a Trauma-Informed Support, Skills, and Psychoeducation Intervention for Survivors of Torture and Related Trauma in Kurdistan, Northern Iraq. Global health, science and practice. 2016;4(3):452-66. doi: <http://dx.doi.org/10.9745/GHSP-D-16-00017>. PubMed PMID: 619970238.

9. Becker ME, Hertzberg MA, Moore SD, Dennis MF, Beckham JC. A placebo-controlled trial of bupropion SR in the treatment of chronic posttraumatic stress disorder. Journal of Clinical Psychopharmacology (USA). 2007;27(2):193-7. doi: 10.1097/JCP.0b013e318032eaed.

10. Beidel DC, Frueh BC, Uhde TW, Wong N, Mentrikoski JM. Multicomponent behavioral treatment for chronic combat-related posttraumatic stress disorder: a randomized controlled trial. Journal of Anxiety Disorders. 2011;25(2):224-31. doi: <https://dx.doi.org/10.1016/j.janxdis.2010.09.006> PubMed PMID: 20951543; PubMed Central PMCID: PMCNIHMS238163.

11. Bichescu D, Neuner F, Schauer M, Elbert T. Narrative exposure therapy for political imprisonment-related chronic posttraumatic stress disorder and depression. Behaviour Research & Therapy. 2007;45(9):2212-20. doi: 10.1016/j.brat.2006.12.006. PubMed PMID: 17288990.

12. Bolton P, Bass JK, Zangana GA, Kamal T, Murray SM, Kaysen D, et al. A randomized controlled trial of mental health interventions for survivors of systematic violence in Kurdistan, Northern Iraq. BMC Psychiatry. 2014;14:360. doi: <https://dx.doi.org/10.1186/s12888-014-0360-2> PubMed PMID: 25551436.

13. Bonilla-Escobar FJ, Fandino-Losada A, Martinez-Buitrago DM, Santaella-Tenorio J, Tobon-Garcia D, Munoz-Morales EJ, et al. A randomized controlled trial of a transdiagnostic cognitive-behavioral intervention for Afro-descendants' survivors of systemic violence in Colombia. PLoS ONE [Electronic Resource]. 2018;13(12):e0208483. doi: <https://dx.doi.org/10.1371/journal.pone.0208483>. PubMed PMID: 30532155.

14. Bremner JD, Mishra S, Campanella C, Shah M, Kasher N, Evans S, et al. A pilot study of the effects of mindfulness-based stress reduction on post-traumatic stress disorder symptoms and brain response to traumatic reminders of combat in Operation Enduring Freedom/Operation Iraqi Freedom combat veterans with post-traumatic stress disorder. Frontiers in Psychiatry Vol 8 2017, ArtID 157. 2017;8. doi: 10.3389/fpsyt.2017.00157. PubMed PMID: 2017-38563-001.

15. Buhmann CB, Nordentoft M, Ekstroem M, Carlsson J, Mortensen EL. The effect of flexible cognitive-behavioural therapy and medical treatment, including antidepressants on post-traumatic stress disorder and depression in traumatised refugees: pragmatic randomised controlled clinical trial. British Journal of Psychiatry. 2016;208(3):252-9. doi: <https://dx.doi.org/10.1192/bjp.bp.114.150961> PubMed PMID: 26541687.

16. Carlson JG, Chemtob CM, Rusnak K, Hedlund NL, Muraoka MY. Eye movement desensitization and reprocessing (EMDR) treatment for combat-related posttraumatic stress disorder. Japanese Journal of Biofeedback Research. 1997;24:50-64. doi: 10.1023/A:1024448814268. PubMed PMID: 1999-13197-003.

17. Carlsson J, Sonne C, Vindbjerg E, Mortensen EL. Stress management versus cognitive restructuring in trauma-affected refugees-A pragmatic randomised study. Psychiatry Research. 2018;266:116-23. doi: <https://dx.doi.org/10.1016/j.psychres.2018.05.015>. PubMed PMID: 29859498.

18. Celik C, Ozdemir B, Ozmenler KN, Yelboga Z, Balikci A, Oznur T, et al. Efficacy of Paroxetine and Amitriptyline in Posttraumatic Stress Disorder: An Open-label Comparative Study. Klinik Psikofarmakoloji Bülteni-Bulletin of Clinical Psychopharmacology. 2011;21(3):179-85. doi: 10.5455/bcp.20110627111141.

19. Chard KM. An evaluation of cognitive processing therapy for the treatment of posttraumatic stress disorder related to childhood sexual abuse. Journal of Consulting & Clinical Psychology. 2005;73(5):965-71. doi: 10.1037/0022-006X.73.5.965. PubMed PMID: 16287396.

20. Chung MY, Min KH, Jun YJ, Kim SS, Kim WC, Jun EM. Efficacy and tolerability of mirtazapine and sertraline in Korean veterans with posttraumatic stress disorder: a randomized open label trial. Human Psychopharmacology. 2004;19(7):489-94. doi: 10.1002/hup.615. PubMed PMID: 15378676.

21. Classen C, Koopman C, Nevillmanning K, Spiegel D. A Preliminary Report Comparing Trauma-Focused and Present-Focused Group Therapy Against a Wait-Listed Condition Among Childhood Sexual Abuse Survivors with PTSD. Journal of Aggression, Maltreatment & Trauma. 2001;4(2):265-88. doi: 10.1300/J146v04n02_12.

22. Cloitre M, Stovall-McClough KC, Nooner K, Zorbas P, Cherry S, Jackson CL, et al. Treatment for PTSD related to childhood abuse: a randomized controlled trial. American Journal of Psychiatry. 2010;167(8):915-24. doi: <https://dx.doi.org/10.1176/appi.ajp.2010.09081247> PubMed PMID: 20595411.

23. Cook JM, Harb GC, Gehrman PR, Cary MS, Gamble GM, Forbes D, et al. Imagery rehearsal for posttraumatic nightmares: a randomized controlled trial. Journal of traumatic stress. 2010;23(5):553-63. doi: 10.1002/jts.20569

24. Davis LL, Jewell ME, Ambrose S, Farley J, English B, Bartolucci A, et al. A Placebo-Controlled Study of Nefazodone for the Treatment of Chronic Posttraumatic Stress Disorder: A Preliminary Study. Journal of Clinical Psychopharmacology. 2004;24(3). doi: 10.1097/01.jcp.0000125685.82219.1a.

25. Davis LL, Davidson JR, Ward LC, Bartolucci A, Bowden CL, Petty F. Divalproex in the treatment of posttraumatic stress disorder: a randomized, double-blind, placebo-controlled trial in a veteran population. Journal of Clinical Psychopharmacology. 2008;28(1):84-8. doi: <https://dx.doi.org/10.1097/JCP.0b013e318160f83b>. PubMed PMID: 18204347.

26. Devilly GJ, Spence SH, Rapee RM. Statistical and reliable change with eye movement desensitization and reprocessing: Treating trauma within a veteran population. Behavior Therapy. 1998;29(3):435-55. doi: <https://doi.org/10.1016/S0005-7894(98)80042-7>.

27. Edmond T, Rubin A, Wambach KG. The effectiveness of EMDR with adult female survivors of childhood sexual abuse. Social Work Research. 1999;23(2):103-16. doi: 10.1093/swr/23.2.103.

28. Engel CC, Litz B, Magruder KM, Harper E, Gore K, Stein N, et al. Delivery of self training and education for stressful situations (DESTRESS-PC): a randomized trial of nurse assisted online self-management for PTSD in primary care. General Hospital Psychiatry. 2015;37(4):323-8. doi: <https://dx.doi.org/10.1016/j.genhosppsych.2015.04.007> PubMed PMID: 25929985; PubMed Central PMCID: PMCNIHMS680713.

29. Feske U. Treating low-income and minority women with posttraumatic stress disorder: a pilot study comparing prolonged exposure and treatment as usual conducted by community therapists. Journal of Interpersonal Violence. 2008;23(8):1027-40. doi: <https://dx.doi.org/10.1177/0886260507313967>. PubMed PMID: 18292398.

30. Foa EB, McLean CP, Zang Y, Rosenfield D, Yadin E, Yarvis JS, et al. Effect of Prolonged Exposure Therapy Delivered Over 2 Weeks vs 8 Weeks vs Present-Centered Therapy on PTSD Symptom Severity in Military Personnel: A Randomized Clinical Trial. JAMA. 2018;319(4):354-64. doi: <https://dx.doi.org/10.1001/jama.2017.21242>. PubMed PMID: 29362795.

31. Ford JD, Grasso DJ, Greene CA, Slivinsky M, DeViva JC. Randomized clinical trial pilot study of prolonged exposure versus present centred affect regulation therapy for PTSD and anger problems with male military combat veterans. Clinical Psychology & Psychotherapy. 2018;25(5):641-9. doi: <https://dx.doi.org/10.1002/cpp.2194>. PubMed PMID: 29687524.

32. Franciskovic T, Sukovic Z, Janovic S, Stevanovic A, Nemcic-Moro I, Roncevic-Grzeta I, et al. Tianeptine in the combined treatment of combat related poasttraumatic stress disorder. Psychiatria Danubina. 2011;23(3):257-63. PubMed PMID: 2011-23671-010.

33. Franklin CL, Cuccurullo LA, Walton JL, Arseneau JR, Petersen NJ. Face to face but not in the same place: A pilot study of prolonged exposure therapy. Journal of Trauma and Dissociation. 2017;18(1):116-30. doi: <http://dx.doi.org/10.1080/15299732.2016.1205704> PubMed PMID: 611575198.

34. Friedman MJ, Marmar CR, Baker DG, Sikes CR, Farfel GM. Randomized, double-blind comparison of sertraline and placebo for posttraumatic stress disorder in a Department of Veterans Affairs setting. Journal of Clinical Psychiatry. 2007;68(5):711-20. doi: 10.4088/jcp.v68n0508. PubMed PMID: 17503980.

35. Galano MM, Grogan-Kaylor AC, Stein SF, Clark HM, Graham-Bermann SA. Posttraumatic stress disorder in Latina women: Examining the efficacy of the Moms' Empowerment Program. Psychological Trauma:Theory, Pesearch, Practice and Policy. 2017;9(3):344-51. doi: <https://dx.doi.org/10.1037/tra0000218>. PubMed PMID: 27869463.

36. Gamito P, Oliveira J, Rosa P, Morais D, Duarte N, OLiverira S, et al. PTSD Elderly War Veterans: A Clinical Controlled Pilot Study. Cyberpsychology, Behavior, and Social Networking. 2010;13(1):43-8. doi: 10.1089/cyber.2009.0237. PubMed PMID: 20528292.

37. Germain A, Richardson R, Moul DE, Mammen O, Haas G, Forman SD, et al. Placebo-controlled comparison of prazosin and cognitive-behavioral treatments for sleep disturbances in US Military Veterans. Journal of Psychosomatic Research. 2012;72(2):89-96. doi: <https://dx.doi.org/10.1016/j.jpsychores.2011.11.010> PubMed PMID: 22281448; PubMed Central PMCID: PMCNIHMS342239.

38. Hamner MB, Faldowski RA, Ulmer HG, Frueh BC, Huber MG, Arana GW. Adjunctive risperidone treatment in post-traumatic stress disorder: a preliminary controlled trial of effects on comorbid psychotic symptoms. International Clinical Psychopharmacology. 2003;18(1):1-8. doi: 10.1097/01.yic.0000050744.67514.6d. PubMed PMID: 12490768.

39. Hermenau K, Hecker T, Schaal S, Maedl A, Elbert T. Addressing post-traumatic stress and aggression by means of narrative exposure: A randomized controlled trial with ex-combatants in the eastern DRC. Journal of Aggression, Maltreatment and Trauma. 2013;22(8):916-34. doi: <http://dx.doi.org/10.1080/10926771.2013.824057> PubMed PMID: 369892869.

40. Hijazi AM, Lumley MA, Ziadni MS, Haddad L, Rapport LJ, Arnetz BB. Brief narrative exposure therapy for posttraumatic stress in Iraqi refugees: a preliminary randomized clinical trial. Journal of Traumatic Stress. 2014;27(3):314-22. doi: <https://dx.doi.org/10.1002/jts.21922> PubMed PMID: 24866253; PubMed Central PMCID: PMCNIHMS595425.

41. Himmerich H, Willmund GD, Zimmermann P, Wolf JE, Buhler AH, Kirkby KC, et al. Serum concentrations of TNF-alphas soluble receptors during psychotherapy in German soldiers suffering from combat-related PTSD. Psychiatria Danubina. 2016;28(3):293-8. doi: 10.4103/0366-6999.178039 PubMed PMID: 612523930.

42. Hinton DE, Pham T, Tran M, Safren SA, Otto MW, Pollack MH. CBT for Vietnamese refugees with treatment-resistant PTSD and panic attacks: a pilot study. Journal of Traumatic Stress. 2004;17(5):429-33. doi: 10.1023/B:JOTS.0000048956.03529.fa. PubMed PMID: 15633922; PubMed Central PMCID: PMCNIHMS144174.

43. Hinton DE, Chhean D, Pich V, Safren SA, Hofmann SG, Pollack MH. A randomized controlled trial of cognitive-behavior therapy for Cambodian refugees with treatment-resistant PTSD and panic attacks: a cross-over design. Journal of Traumatic Stress. 2005;18(6):617-29. doi: 10.1002/jts.20070. PubMed PMID: 16382423.

44. Jensen JA. An investigation of eye movement desensitization and reprocessing (EMD/R) as a treatment for posttraumatic stress disorder (PTSD) symptoms of Vietnam combat veterans. Behavior Therapy. 1994;25(2):311-25. doi: 10.1016/S0005-7894(05)80290-4. PubMed PMID: 1994-42215-001.

45. Jung K, Steil R. A randomized controlled trial on cognitive restructuring and imagery modification to reduce the feeling of being contaminated in adult survivors of childhood sexual abuse suffering from posttraumatic stress disorder. Psychotherapy & Psychosomatics. 2013;82(4):213-20. doi: <https://dx.doi.org/10.1159/000348450> PubMed PMID: 23712073.

46. Katz LS, Douglas S, Zaleski K, Williams J, Huffman C, Cojucar G. Comparing holographic reprocessing and prolonged exposure for women veterans with sexual trauma: A pilot randomized trial. Journal of Contemporary Psychotherapy. 2014;44(1):9-19. doi: <http://dx.doi.org/10.1007/s10879-013-9248-6> PubMed PMID: 52756441.

47. Keane TM, Fairbank JA, Caddell JM, Zimering RT. Implosive (flooding) therapy reduces symptoms of PTSD in Vietnam combat veterans. Behavior Therapy. 1989;20(2):245-60. doi: <https://doi.org/10.1016/S0005-7894(89)80072-3>.

48. Kearney DJ, McDermott K, Malte C, Martinez M, Simpson TL. Effects of participation in a mindfulness program for veterans with posttraumatic stress disorder: a randomized controlled pilot study. Journal of Clinical Psychology. 2013;69(1):14-27. doi: <https://dx.doi.org/10.1002/jclp.21911> PubMed PMID: 22930491.

49. King AP, Erickson TM, Giardino ND, Favorite T, Rauch SA, Robinson E, et al. A pilot study of group mindfulness-based cognitive therapy (MBCT) for combat veterans with posttraumatic stress disorder (PTSD). Depression & Anxiety. 2013;30(7):638-45. doi: <https://dx.doi.org/10.1002/da.22104> PubMed PMID: 23596092; PubMed Central PMCID: PMCNIHMS671148.

50. Knaevelsrud C, Brand J, Lange A, Ruwaard J, Wagner B. Web-based psychotherapy for posttraumatic stress disorder in war-traumatized Arab patients: randomized controlled trial. Journal of Medical Internet Research. 2015;17(3):e71. doi: <https://dx.doi.org/10.2196/jmir.3582> PubMed PMID: 25799024.

51. Knaevelsrud C, Böttche M, Pietrzak RH, Freyberger HJ, Kuwert P. Efficacy and Feasibility of a Therapist-Guided Internet-Based Intervention for Older Persons with Childhood Traumatization: A Randomized Controlled Trial. American Journal of Geriatric Psychiatry. 2017;25(8):878-88. doi: 10.1016/j.jagp.2017.02.024. PubMed PMID: 124186984.

52. Kosten TR, Krystal JH, Giller EL, Frank J, Dan E. Alexithymia as a predictor of treatment response in post-traumatic stress disorder. Journal of Traumatic Stress. 1992;5(4):563-73. doi: 10.1016/j.biopsych.2013.05.017. PubMed PMID: 1993-18480-001.

53. Krupnick JL, Green BL, Stockton P, Miranda J, Krause E, Mete M. Group interpersonal psychotherapy for low-income women with posttraumatic stress disorder. Psychotherapy Research. 2008;18(5):497-507. doi: <https://dx.doi.org/10.1080/10503300802183678>. PubMed PMID: 18816001.

54. Kruse J, Joksimovic L, Cavka M, Woller W, Schmitz N. Effects of trauma-focused psychotherapy upon war refugees. Journal of Traumatic Stress. 2009;22(6):585-92. doi: <https://dx.doi.org/10.1002/jts.20477> PubMed PMID: 19960519.

55. Krystal JH, Rosenheck RA, Cramer JA, Vessicchio JC, Jones KM, Vertrees JE, et al. Adjunctive risperidone treatment for antidepressant-resistant symptoms of chronic military service-related PTSD: a randomized trial. JAMA. 2011;306(5):493-502. doi: <https://dx.doi.org/10.1001/jama.2011.1080> PubMed PMID: 21813427.

56. Kubany ES, Hill EE, Owens JA. Cognitive trauma therapy for battered women with PTSD: preliminary findings. Journal of Traumatic Stress. 2003;16(1):81-91. doi: 10.1023/A:1022019629803. PubMed PMID: 12602656.

57. Kubany ES, Hill EE, Owens JA, Iannce-Spencer C, McCaig MA, Tremayne KJ, et al. Cognitive trauma therapy for battered women with PTSD (CTT-BW). Journal of Consulting & Clinical Psychology. 2004;72(1):3-18. doi: <https://dx.doi.org/10.1037/0022-006X.72.1.3> PubMed PMID: 14756610.

58. Lande RG, Williams LB, Francis JL, Gragnani C, Morin ML. Efficacy of biofeedback for post-traumatic stress disorder. Complementary Therapies in Medicine. 2010;18(6):256-9. doi: <https://dx.doi.org/10.1016/j.ctim.2010.08.004> PubMed PMID: 21130362.

59. Lau M, Kristensen E. Outcome of systemic and analytic group psychotherapy for adult women with history of intrafamilial childhood sexual abuse: a randomized controlled study. Acta Psychiatrica Scandinavica. 2007;116(2):96-104. doi: 10.1111/j.1600-0447.2006.00977.x. PubMed PMID: WOS:000248087900003.

60. Levi O, Bar-Haim Y, Kreiss Y, Fruchter E. Cognitive-behavioural therapy and psychodynamic psychotherapy in the treatment of combat-related post-traumatic stress disorder: a comparative effectiveness study. Clinical Psychology & Psychotherapy. 2016;23(4):298-307. doi: 10.1002/cpp.1969 PubMed PMID: 117169055.

61. Lindley SE, Carlson EB, Hill K. A randomized, double-blind, placebo-controlled trial of augmentation topiramate for chronic combat-related posttraumatic stress disorder. Journal of Clinical Psychopharmacology. 2007;27(6):677-81. doi: <https://dx.doi.org/10.1097/jcp.0b013e31815a43ee>. PubMed PMID: 18004136.

62. Lundqvist G, Svedin CG, Hansson K, Broman I. Group therapy for women sexually abused as children: mental health before and after group therapy. Journal of Interpersonal Violence. 2006;21(12):1665-77. doi: 10.1177/0886260506294986. PubMed PMID: 17065660.

63. Margolies SO, Rybarczyk B, Lynch J, Vrana S. Efficacy of a cognitive-behavioral treatment for insomnia among Afghanistan and Iraq (OEF/ OIF) veterans with PTSD. Sleep. 2011;34:A253-A4. doi: 0.1002/jclp.21970. PubMed PMID: 71510995.

64. McDonagh A, Friedman M, McHugo G, Ford J, Sengupta A, Mueser K, et al. Randomized trial of cognitive-behavioral therapy for chronic posttraumatic stress disorder in adult female survivors of childhood sexual abuse. Journal of Consulting & Clinical Psychology. 2005;73(3):515-24. doi: 10.1037/0022-006X.73.3.515. PubMed PMID: 15982149.

65. McLay RN, Wood DP, Webb-Murphy JA, Spira JL, Wiederhold MD, Pyne JM, et al. A randomized, controlled trial of virtual reality-graded exposure therapy for post-traumatic stress disorder in active duty service members with combat-related post-traumatic stress disorder. Cyberpsychology, behavior and social networking. 2011;14(4):223-9. doi: <https://dx.doi.org/10.1089/cyber.2011.0003> PubMed PMID: 21332375.

66. Meffert SM, Abdo AO, Alla OAA, Elmakki YOM, Omer AA, Yousif S, et al. A pilot randomized controlled trial of interpersonal psychotherapy for Sudanese refugees in Cairo, Egypt. Psychological Trauma: Theory, Research, Practice, and Policy. 2014;6(3):240-9. doi: 10.1037/a0023540.

67. Miyahira SD, Folen RA, Hoffman HG, Garcia-Palacios A, Spira JL, Kawasaki M. The effectiveness of VR exposure therapy for PTSD in returning warfighters. Annual Review of CyberTherapy and Telemedicine. 2012;10:128-32. doi: 10.3233/978-1-61499-121-2-128.

68. Monnelly EP, Ciraulo DA, Knapp C, Keane T. Low-dose risperidone as adjunctive therapy for irritable aggression in posttraumatic stress disorder. Journal of Clinical Psychopharmacology. 2003;23(2):193-6. PubMed PMID: 12640221.

69. Moradi AR, Moshirpanahi S, Parhon H, Mirzaei J, Dalgleish T, Jobson L. A pilot randomized controlled trial investigating the efficacy of MEmory Specificity Training in improving symptoms of posttraumatic stress disorder. Behaviour Research & Therapy. 2014;56:68-74. doi: <https://dx.doi.org/10.1016/j.brat.2014.03.002> PubMed PMID: 24705337.

70. Morgan T, Cummings AL. Change experienced during group therapy by female survivors of childhood sexual abuse. Journal of Consulting and Clinical Psychology. 1999;67(1):28-36. doi: 10.1037/0022-006X.67.1.28.

71. Nakamura Y, Lipschitz DL, Donaldson GW, Kida Y, Williams SL, Landward R, et al. Investigating Clinical Benefits of a Novel Sleep-Focused Mind-Body Program on Gulf War Illness Symptoms: A Randomized Controlled Trial. Psychosomatic Medicine. 2017;79(6):706-18. doi: <https://dx.doi.org/10.1097/PSY.0000000000000469>. PubMed PMID: 28406803.

72. Narimani M, Sadeghieh Ahari S, Rajabi S. Comparison of efficacy of eye movement desensitization and reprocessing and cognitive behavioral therapy therapeutic methods for reducing anxiety and depression of Iranian combatant afflicted by post traumatic stress disorder. Journal of Applied Sciences. 2008;8(10):1932-7. doi: <http://dx.doi.org/10.3923/jas.2008.1932.1937>

73. Naylor JC, Dolber TR, Strauss JL, Kilts JD, Strauman TJ, Bradford DW, et al. A pilot randomized controlled trial with paroxetine for subthreshold PTSD in Operation Enduring Freedom/Operation Iraqi Freedom era veterans. Psychiatry Research. 2013;206(2-3):318-20. doi: 10.1016/j.psychres.2012.11.008. PubMed PMID: 2013-00092-001.

74. Neuner F, Onyut PL, Ertl V, Odenwald M, Schauer E, Elbert T. Treatment of posttraumatic stress disorder by trained lay counselors in an African refugee settlement: a randomized controlled trial. Journal of Consulting & Clinical Psychology. 2008;76(4):686-94. doi: <https://dx.doi.org/10.1037/0022-006X.76.4.686> PubMed PMID: 18665696.

75. Neuner F, Schauer M, Klaschik C, Karunakara U, Elbert T. A comparison of narrative exposure therapy, supportive counseling, and psychoeducation for treating posttraumatic stress disorder in an African refugee settlement. Journal of Consulting & Clinical Psychology. 2004;72(4):579-87. doi: 10.1037/0022-006X.72.4.579. PubMed PMID: 15301642.

76. Niles BL, Klunk-Gillis J, Ryngala DJ, Silberbogen AK, Paysnick A, EJ. W. Comparing mindfulness and psychoeducation treatments for combat-related PTSD using a telehealth approach. Psychological Trauma: Theory, Research, Practice, and Policy. 2012;4:538-47. doi: 10.1037/a0026161.

77. Otto MW, Hinton D, Korbly NB, Chea A, Ba P, Gershuny BS, et al. Treatment of pharmacotherapy-refractory posttraumatic stress disorder among Cambodian refugees: a pilot study of combination treatment with cognitive-behavior therapy vs sertraline alone. Behav Res Ther. 2003;41(11):1271-6. doi: 10.1016/s0005-7967(03)00032-9. PubMed PMID: 14527527.

78. Owens GP, Pike JL, Chard KM. Treatment effects of cognitive processing therapy on cognitive distortions of female child sexual abuse survivors. Behavior Therapy. 2001;32(3):413-24. doi: <https://doi.org/10.1016/S0005-7894(01)80028-9>. PubMed PMID: 2002-12369-001.

79. Panahi Y, Moghaddam BR, Sahebkar A, Nazari MA, Beiraghdar F, Karami G, et al. A randomized, double-blind, placebo-controlled trial on the efficacy and tolerability of sertraline in Iranian veterans with post-traumatic stress disorder. Psychological Medicine. 2011;41(10):2159-66. doi: <https://dx.doi.org/10.1017/S0033291711000201> PubMed PMID: 21349225.

80. Paunovic N, Ost LG. Cognitive-behavior therapy vs exposure therapy in the treatment of PTSD in refugees. Behav Res Ther. 2001;39(10):1183-97. doi: 10.1016/S0005-7967(00)00093-0. PubMed PMID: 11579988.

81. Pivac N, Kozaric-Kovacic D, Muck-Seler D. Olanzapine versus fluphenazine in an open trial in patients with psychotic combat-related post-traumatic stress disorder. Psychopharmacology. 2004;175(4):451-6. doi: 10.1007/s00213-004-1849-z. PubMed PMID: 2004-19344-008.

82. Polusny MA, Erbes CR, Thuras P, Moran A, Lamberty GJ, Collins RC, et al. Mindfulness-based stress reduction for posttraumatic stress disorder among veterans: a randomized clinical trial. JAMA. 2015;314(5):456-65. doi: <https://dx.doi.org/10.1001/jama.2015.8361> PubMed PMID: 26241597.

83. Possemato K, Bergen-Cico D, Treatman S, Allen C, Wade M, Pigeon W. A randomized clinical trial of primary care brief mindfulness training for veterans with PTSD. Journal of Clinical Psychology. 2016;72(3):179-93. doi: <https://dx.doi.org/10.1002/jclp.22241> PubMed PMID: 26613203.

84. Raskind MA, Peskind ER, Hoff DJ, Hart KL, Holmes HA, Warren D, et al. A parallel group placebo controlled study of prazosin for trauma nightmares and sleep disturbance in combat veterans with post-traumatic stress disorder. Biological Psychiatry. 2007;61(8):928-34. doi: <https://dx.doi.org/10.1016/j.biopsych.2006.06.032>. PubMed PMID: 17069768.

85. Raskind MA, Peterson K, Williams T, Hoff DJ, Peskind ER. A trial of prazosin for combat trauma PTSD with nightmares in active-duty soldiers returned from Iraq and Afghanistan. American Journal of Psychiatry. 2013;170(9):1003-10. doi: 10.1176/appi.ajp.2013.12081133.

86. Rauch SA, Kim H, Powell C, Tuerk PW, Simon NM, Acierno R, et al. Efficacy of prolonged exposure therapy, sertraline hydrochloride, and their combination among combat veterans with posttraumatic stress disorder: A randomized clinical trial. JAMA Psychiatry. 2019;76(2):117-25. doi: <http://dx.doi.org/10.1001/jamapsychiatry.2018.3412>. PubMed PMID: 2019-07144-001.

87. Ready DJ, Gerardi RJ, Backscheider AG, Mascaro N, Rothbaum BO. Comparing virtual reality exposure therapy to present-centered therapy with 11 U.S. Vietnam veterans with PTSD. Cyberpsychol Behav Soc Netw. 2010;13(1):49-54. doi: 10.1089/cyber.2009.0239. PubMed PMID: 20528293.

88. Reed GL, Enright RD. The effects of forgiveness therapy on depression, anxiety, and posttraumatic stress for women after spousal emotional abuse. Journal of Consulting & Clinical Psychology. 2006;74(5):920-9. doi: <https://dx.doi.org/10.1037/0022-006X.74.5.920>. PubMed PMID: 17032096.

89. Reger GM, Koenen-Woods P, Zetocha K, Smolenski DJ, Holloway KM, Rothbaum BO, et al. Randomized controlled trial of prolonged exposure using imaginal exposure vs. virtual reality exposure in active duty soldiers with deployment-related posttraumatic stress disorder (PTSD). Journal of Consulting and Clinical Psychology. 2016;84(11):946-59. doi: <http://dx.doi.org/10.1037/ccp0000134> PubMed PMID: 612057864.

90. Reich DB, Winternitz S, Hennen J, Watts T, Stanculescu C. A preliminary study of risperidone in the treatment of posttraumatic stress disorder related to childhood abuse in women. Journal of Clinical Psychiatry. 2004;65(12):1601-6. doi: 0.4088/jcp.v65n1204. PubMed PMID: 15641864.

91. Resick PA, Wachen JS, Mintz J, Young-McCaughan S, Roache JD, Borah AM, et al. A randomized clinical trial of group cognitive processing therapy compared with group present-centered therapy for PTSD among active duty military personnel. Journal of Consulting & Clinical Psychology. 2015;83(6):1058-68. doi: <https://dx.doi.org/10.1037/ccp0000016> PubMed PMID: 25939018.

92. Rogers S, Silver SM, Goss J, Obenchain J, Willis A, Whitney RL. A Single Session, Group Study of Exposure and Eye Movement Desensitization and Reprocessing in Treating Posttraumatic Stress Disorder Among Vietnam War Veterans: Preliminary Data. J Anxiety Disord. 1999;13(1):119-30. doi: <https://doi.org/10.1016/S0887-6185(98)00043-7>.

93. Salo J, Punamaki R-L, Qouta S, El Sarraj E. Individual and group treatment and self and other representations predicting posttraumatic recovery among former political prisoners. Traumatology. 2008;14(2):45-61. doi: 10.1177/1534765608319079. PubMed PMID: 2009-10421-005.

94. Saxe BJ, Johnson SM. An empirical investigation of group treatment for a clinical population of adult female incest survivors. Journal of Child Sexual Abuse. 1999;8(1):67-88. doi: <https://doi.org/10.1300/J070v08n01_05>. PubMed PMID: 107099962.

95. Shapiro E, Laub B, Rosenblat O. Early EMDR intervention following intense rocket attacks on a town: A randomised clinical trial. Clinical Neuropsychiatry: Journal of Treatment Evaluation. 2018;15(3):194-205. doi: 10.1080/20008198.2019.1695486. PubMed PMID: 2018-27261-003.

96. Sikkema KJ, Hansen NB, Kochman A, Tarakeshwar N, Neufeld S, Meade CS, et al. Outcomes from a group intervention for coping with HIV/AIDS and childhood sexual abuse: reductions in traumatic stress. AIDS & Behavior. 2007;11(1):49-60. doi: <https://dx.doi.org/10.1007/s10461-006-9149-8>. PubMed PMID: 16858634.

97. Sikkema KJ, Ranby KW, Meade CS, Hansen NB, Wilson PA, Kochman A. Reductions in traumatic stress following a coping intervention were mediated by decreases in avoidant coping for people living with HIV/AIDS and childhood sexual abuse. Journal of Consulting & Clinical Psychology. 2013;81(2):274-83. doi: <https://dx.doi.org/10.1037/a0030144> PubMed PMID: 23025248; PubMed Central PMCID: PMCNIHMS433358.

98. Smajkic A, Weine S, Djuric-Bijedic Z, Boskailo E, Lewis J, Pavkovic I. Sertraline, paroxetine, and venlafaxine in refugee posttraumatic stress disorder with depression symptoms. Journal of Traumatic Stress. 2001;14(3):445-52. doi: <https://dx.doi.org/10.1023/A:1011177420069>. PubMed PMID: 11534876.

99. Sonne C, Carlsson J, Bech P, Elklit A, Mortensen EL. Treatment of trauma-affected refugees with venlafaxine versus sertraline combined with psychotherapy-A randomised study. BMC Psychiatry Vol 16 2016, ArtID 383. 2016;16. doi: 10.1186/s12888-016-1081-5. PubMed PMID: 2016-54614-001.

100. Spidel A, Lecomte T, Kealy D, Daigneault I. Acceptance and commitment therapy for psychosis and trauma: Improvement in psychiatric symptoms, emotion regulation, and treatment compliance following a brief group intervention. Psychology and Psychotherapy-Theory Research and Practice. 2018;91(2):248-61. doi: 10.1111/papt.12159. PubMed PMID: WOS:000434168200007.

101. Stein MB, Kline NA, Matloff JL. Adjunctive olanzapine for SSRI-resistant combat-related PTSD: A double-blind, placebo-controlled study. The American Journal of Psychiatry. 2002;159(10):1777-9. doi: 10.1176/appi.ajp.159.10.1777. PubMed PMID: 2002-04602-025.

102. Stenmark H, Catani C, Neuner F, Elbert T, Holen A. Treating PTSD in refugees and asylum seekers within the general health care system. A randomized controlled multicenter study. Behaviour Research & Therapy. 2013;51(10):641-7. doi: <https://dx.doi.org/10.1016/j.brat.2013.07.002> PubMed PMID: 23916633.

103. Teng EJ, Bailey SD, Chaison AD, Petersen NJ, Hamilton JD, Dunn NJ. Treating comorbid panic disorder in veterans with posttraumatic stress disorder. Journal of Consulting & Clinical Psychology. 2008;76(4):704-10. doi: <https://dx.doi.org/10.1037/0022-006X.76.4.710> PubMed PMID: 18665698.

104. Ter Heide FJ, Mooren TM, Kleijn W, de Jongh A, Kleber RJ. EMDR versus stabilisation in traumatised asylum seekers and refugees: results of a pilot study. European Journal of Psychotraumatology. 2011;2. doi: <https://dx.doi.org/10.3402/ejpt.v2i0.5881> PubMed PMID: 22893808.

105. Ter Heide FJ, Mooren TM, van de Schoot R, de Jongh A, Kleber RJ. Eye movement desensitisation and reprocessing therapy v. stabilisation as usual for refugees: randomised controlled trial. British Journal of Psychiatry. 2016;209(4):311-8. doi: <https://dx.doi.org/10.1192/bjp.bp.115.167775> PubMed PMID: 26892849.

106. Thorp SR, Glassman LH, Wells SY, Walter KH, Gebhardt H, Twamley E, et al. A randomized controlled trial of prolonged exposure therapy versus relaxation training for older veterans with military-related PTSD. Journal of Anxiety Disorders. 2019;64:45-54. doi: <http://dx.doi.org/10.1016/j.janxdis.2019.02.003>. PubMed PMID: 2019-27063-007.

107. Ulmer CS, Edinger JD, Calhoun PS. A multi-component cognitive-behavioral intervention for sleep disturbance in veterans with PTSD: a pilot study. J Clin Sleep Med. 2011;7(1):57-68. doi: 10.5664/jcsm.28042 PubMed PMID: 21344046.

108. Van der Kolk BA, Dreyfuss D, Michaels M, Shera D, Berkowitz R, Fisler R, et al. Fluoxetine in posttraumatic stress disorder. Journal of Clinical Psychiatry. 1994;55(12):517-22. PubMed PMID: 25026437.

109. Wahbeh H, Goodrich E, Goy E, Oken BS. Mechanistic pathways of mindfulness meditation in combat veterans with posttraumatic stress disorder. Journal of Clinical Psychology. 2016;72(4):365-83. doi: <https://dx.doi.org/10.1002/jclp.22255> PubMed PMID: 26797725; PubMed Central PMCID: PMCNIHMS744049 [Available on 04/01/17].

110. Wang SJ, Bytyci A, Izeti S, Kallaba M, Rushiti F, Montgomery E, et al. A novel bio-psycho-social approach for rehabilitation of traumatized victims of torture and war in the post-conflict context: a pilot randomized controlled trial in Kosovo. Conflict & Health [Electronic Resource]. 2016;10:34. doi: <https://dx.doi.org/10.1186/s13031-016-0100-y> PubMed PMID: 28191034.

111. Weiss WM, Murray LK, Zangana GA, Mahmooth Z, Kaysen D, Dorsey S, et al. Community-based mental health treatments for survivors of torture and militant attacks in Southern Iraq: a randomized control trial. BMC Psychiatry. 2015;15:249. doi: <https://dx.doi.org/10.1186/s12888-015-0622-7> PubMed PMID: 26467303.

112. Yeomans PD, Forman EM, Herbert JD, Yuen EK. A randomized trial of a reconciliation workshop with and without PTSD psychoeducation in Burundian sample. Journal of Traumatic Stress. 2010;23(3):305-12. doi: <http://dx.doi.org/10.1002/jts.20531>

113. Yurtsever A, Konuk E, Akyuz T, Zat Z, Tukel F, Cetinkaya M, et al. An Eye Movement Desensitization and Reprocessing (EMDR) Group Intervention for Syrian Refugees With Post-traumatic Stress Symptoms: Results of a Randomized Controlled Trial. Frontiers in Psychology. 2018;9:493. doi: <https://dx.doi.org/10.3389/fpsyg.2018.00493>. PubMed PMID: 29946275.

114. Zlotnick C, Shea TM, Rosen K, Simpson E, Mulrenin K, Begin A, et al. An affect-management group for women with posttraumatic stress disorder and histories of childhood sexual abuse. Journal of Traumatic Stress. 1997;10(3):425-36. doi: 10.1023/a:1024841321156. PubMed PMID: 9246650.

115. Zohar J, Amital D, Miodownik C, Kotler M, Bleich A, Lane RM, et al. Double-blind placebo-controlled pilot study of sertraline in military veterans with posttraumatic stress disorder. Journal of Clinical Psychopharmacology. 2002;22(2):190-5. doi: 10.1097/00004714-200204000-00013. PubMed PMID: 11910265.
