# Supplementary material for: Psychological and pharmacological interventions for posttraumatic stress disorder and comorbid mental health problems following complex traumatic events: Systematic review and component network meta-analysis
Source: PLoS Med. 2020 Aug 19;17(8):e1003262. doi: 10.1371/journal.pmed.1003262 (PMC7446790; doi:10.1371/journal.pmed.1003262)
Supplement: S2 Table — (DOCX) [file pmed.1003262.s004.docx]

S2 Table Risk of bias assessments for randomised controlled trials

| **Study** | Random sequence generation | Allocation concealment | Blinding of participants and personnel | Blinding of outcome assessment | Incomplete outcome data | Selective reporting | Other bias |
| --- | --- | --- | --- | --- | --- | --- | --- |
|  | Selection bias | Selection bias | Performance bias | Detection bias | Attrition bias | Reporting bias |  |
| Acarturk et al[1] | Low | High | High | Low | Uncertain | Uncertain | Uncertain |
| Acarturk et al[2] | Low | High | High | Low | Low | Uncertain | Uncertain |
| Adenauer et al[3] | Low | Uncertain | High | Low | High | High | Uncertain |
| Ardani et al[4] | Uncertain | Low | Low | Uncertain | Low | High | Uncertain |
| Azad et al[5] | Uncertain | Uncertain | Uncertain | Uncertain | High | Uncertain | Uncertain |
| Bahadir-Yilmaz et al[6] | Uncertain | Uncertain | High | Uncertain | High | Uncertain | Uncertain |
| Bartozkis et al[7] | Uncertain | Uncertain | Low | Uncertain | High | Uncertain | Uncertain |
| Bass et al[8] | Low | Low | High | Low | Low | Uncertain | Uncertain |
| Becker et al[9] | Uncertain | Uncertain | Low | Low | Uncertain | Uncertain | Uncertain |
| Beidel et al[10] | Uncertain | Uncertain | High | Uncertain | Uncertain | Uncertain | Uncertain |
| Bichescu et al[11] | Uncertain | Uncertain | High | High | Uncertain | Uncertain | Uncertain |
| Bolton et al[12] | Low | Uncertain | High | Low | Low | Uncertain | Uncertain |
| Bonillar-Escobar et al[13] | Low | Uncertain | High | Uncertain | Low | Uncertain | Uncertain |
| Bremner et al[14] | Uncertain | Uncertain | High | Uncertain | High | Uncertain | Uncertain |
| Buhmann et al[15] | Low | Low | High | Low | Low | Low | Uncertain |
| Carlson et al[16] | Uncertain | Uncertain | High | Uncertain | Uncertain | Uncertain | Uncertain |
| Carlson et al[17] | Low | Low | High | High | Low | Uncertain | Uncertain |
| Celik et al[18] | Uncertain | Uncertain | High | High | Uncertain | Uncertain | Uncertain |
| Chard et al[19] | Uncertain | High | High | Low | Uncertain | Uncertain | Uncertain |
| Chung et al[20] | Uncertain | Uncertain | Uncertain | Uncertain | High | Uncertain | Low |
| Classen et al[21] | Uncertain | High | High | Uncertain | Low | Uncertain | Low |
| Cloitre et al[22] | Uncertain | Uncertain | High | Uncertain | High | Uncertain | Low |
| Cook et al[23] | Low | Uncertain | Uncertain | Low | Low | Uncertain | Low |
| Davis et al[24] | Uncertain | Uncertain | Low | Uncertain | Uncertain | Uncertain | Low |
| Davis et al[25] | Low | Low | Low | Low | Uncertain | Uncertain | Low |
| Devilly et al[26] | Uncertain | Uncertain | High | High | Uncertain | Uncertain | Low |
| Edmond et al[27] | Uncertain | Uncertain | High | Uncertain | Low | Uncertain | Low |
| Engel et al[28] | Low | Low | High | Low | Low | High | Low |
| Feske et al[29] | Uncertain | Uncertain | Uncertain | Uncertain | High | Uncertain | Low |
| Foa et al[30] | Low | Uncertain | High | Low | Low | Low | Uncertain |
| Ford et al[31] | Uncertain | Low | Uncertain | Low | Uncertain | Low | Uncertain |
| Franciskovic et al[32] | Uncertain | Uncertain | Uncertain | Uncertain | High | Uncertain | Low |
| Franklin et al[33] | Uncertain | Uncertain | High | Low | High | Uncertain | Low |
| Friedman et al[34] | Low | Uncertain | Low | Low | Low | Uncertain | Low |
| Galano et al[35] | Uncertain | High | High | High | Uncertain | Uncertain | Uncertain |
| Gamito et al[36] | Uncertain | Uncertain | High | Uncertain | High | Uncertain | Low |
| Germain et al[37] | Uncertain | Uncertain | Uncertain | Uncertain | High | Uncertain | Low |
| Hamner et al[38] | Uncertain | Uncertain | Low | Low | Low | Uncertain | Low |
| Hermenau et al[39] | Low | Uncertain | Uncertain | Low | High | Uncertain | Low |
| Hijazi et al[40] | Low | Low | High | High | Low | Uncertain | Low |
| Himmerich et al[41] | Uncertain | Uncertain | High | Uncertain | Uncertain | Uncertain | Low |
| Hinton et al[42] | Uncertain | Uncertain | High | Uncertain | Uncertain | Uncertain | Low |
| Hinton et al[43] | Low | High | Uncertain | Low | Uncertain | High | Low |
| Jensen et al[44] | Uncertain | High | High | Uncertain | High | Uncertain | Low |
| Jung et al[45] | Uncertain | Uncertain | Uncertain | Uncertain | Low | Uncertain | Low |
| Katz et al[46] | Uncertain | Uncertain | High | Uncertain | High | Uncertain | Low |
| Keane et al[47] | Uncertain | Uncertain | High | High | Uncertain | High | Low |
| Kearney et al[48] | Uncertain | Low | High | Uncertain | Uncertain | Low | Low |
| Knaevelsrud et al[49] | Low | Uncertain | High | Uncertain | Uncertain | Uncertain | Low |
| Knaevelsrud et al[50] | Low | Uncertain | High | High | Low | Low | Uncertain |
| Kosten et al[51] | Low | Uncertain | Uncertain | Uncertain | Uncertain | Uncertain | Low |
| Krupnick et al[52] | Uncertain | Uncertain | High | Uncertain | Low | Uncertain | Low |
| Krystal et al[53] | Low | Low | Low | Low | Low | Low | Low |
| Kubany et al[54] | Uncertain | Uncertain | High | Uncertain | High | Uncertain | High |
| Kubany et al[55] | Uncertain | High | High | Low | Uncertain | Uncertain | Low |
| Lande et al[56] | High | High | High | Uncertain | High | Uncertain | Low |
| Lau et al[57] | Low | Uncertain | Low | Uncertain | Uncertain | Uncertain | Low |
| Lindley et al[58] | Uncertain | Uncertain | High | Low | High | Uncertain | Low |
| Lundqvist et al[59] |  |  |  |  |  |  |  |
| Margolies et al[60] | Uncertain | Uncertain | High | Uncertain | High | Uncertain | Low |
| McDonagh et al[61] | Uncertain | Uncertain | Uncertain | Low | Uncertain | High | Low |
| McLay et al[62] | High | High | High | High | High | Uncertain | Uncertain |
| Meffert et al[63] | Low | High | High | High | High | Uncertain | Low |
| Miyahira et al[64] | Uncertain | Uncertain | Uncertain | Uncertain | High | Uncertain | Low |
| Monnelly et al[65] | Uncertain | Uncertain | Uncertain | Uncertain | Uncertain | High | Low |
| Moradi et al[66] | Low | Uncertain | Uncertain | Uncertain | Low | Uncertain | Low |
| Nakamura et al[67] | Low | Uncertain | High | Low | Low | Low | Uncertain |
| Narimani et al[68] |  |  |  |  |  |  |  |
| Naylor et al[69] | Uncertain | Uncertain | Uncertain | Uncertain | Uncertain | Uncertain | Low |
| Neuner et al[70] | Low | High | Uncertain | Low | Low | Uncertain | Low |
| Neuner et al[71] | Uncertain | High | High | Low | High | Uncertain | Low |
| Niles et al[72] | Low | Low | High | High | High | Uncertain | Low |
| Otto et al[73] | Uncertain | Uncertain | High | Uncertain | Low | High | Uncertain |
| Owens et al[74] | Uncertain | Uncertain | High | Uncertain | Uncertain | High | Low |
| Panahi et al[75] | Low | Uncertain | Low | Low | Uncertain | Uncertain | Low |
| Paunovic et al[76] | Uncertain | Uncertain | High | Uncertain | High | High | Low |
| Polusny et al[77] | Low | Uncertain | High | High | Low | Low | Low |
| Possemato et al[78] | Low | Uncertain | Uncertain | Low | Low | High | Low |
| Raskind et al[79] | Low | Low | Low | Low | Uncertain | Uncertain | Low |
| Raskind et al[80] | Uncertain | Uncertain | Low | Low | High | Uncertain | Low |
| Rauch et al[81] | Low | Low | Low | Low | Low | Low | Low |
| Ready et al[82] | Uncertain | Uncertain | High | Low | High | Uncertain | Low |
| Reed et al[83] | Uncertain | Uncertain | Uncertain | Uncertain | Uncertain | High | Low |
| Reger et al[84] | Low | Uncertain | Uncertain | High | Low | Low | Low |
| Reich et al[85] | Uncertain | Uncertain | Uncertain | Uncertain | Low | Uncertain | Low |
| Resick et al[86] | Uncertain | Uncertain | High | Low | High | Uncertain | Low |
| Rogers et al[87] | Uncertain | Uncertain | High | Low | Low | Uncertain | Low |
| Shapiro et al[88] | Low | Low | High | Low | High | Uncertain | Uncertain |
| Sikkema et al[89] | Uncertain | Uncertain | Uncertain | Uncertain | Low | Uncertain | Low |
| Sikkema et al[90] | Uncertain | Uncertain | Uncertain | Uncertain | Low | Uncertain | Low |
| Smajkic et al[91] | Uncertain | Uncertain | Uncertain | Uncertain | High | High | Low |
| Sonne et al[92] | Low | Low | High | Low | Uncertain | High | Low |
| Spidel et al[93] | Uncertain | High | High | Uncertain | Low | Uncertain | Uncertain |
| Stein et al[94] | Uncertain | Uncertain | Low | Low | Uncertain | Uncertain | Low |
| Stenmark et al[95] | Low | Uncertain | High | Low | High | High | Low |
| Teng et al[96] | Low | High | Uncertain | Uncertain | Uncertain | Uncertain | Low |
| Ter Heide et al[97] | Uncertain | High | High | Uncertain | High | Uncertain | Low |
| Ter Heide et al[98] | Low | High | Uncertain | Uncertain | High | Low | Low |
| Thorp et al[99] | Uncertain | Uncertain | Uncertain | Uncertain | Uncertain | Low | Uncertain |
| Ulmer et al[100] | Uncertain | Uncertain | High | Uncertain | Low | Uncertain | Low |
| Van der Kolk et al[101] | Uncertain | Uncertain | Low | Uncertain | High | Uncertain | Low |
| Wahbeh et al[102] | Low | Uncertain | High | Low | High | Uncertain | Low |
| Wang et al[103] | Low | Low | Low | Low | High | Uncertain | Low |
| Weiss et al trial 1: CETA[104] | Low | Uncertain | High | Low | Low | High | Low |
| Yeomans et al[105] | Low | Uncertain | High | High | High | Uncertain | Low |
| Yurtsever et al[106] | Low | Uncertain | High | Low | High | Uncertain | Uncertain |
| Zlotnick et al[107] | Uncertain | Uncertain | High | High | High | Uncertain | Low |
| Zohar et al[108] | Uncertain | Uncertain | Uncertain | Uncertain | High | High | Low |

References

1. Acarturk C, Konuk E, Cetinkaya M, Senay I, Sijbrandij M, Cuijpers P, et al. EMDR for Syrian refugees with posttraumatic stress disorder symptoms: results of a pilot randomized controlled trial. European Journal of Psychotraumatology. 2015;6:27414. doi: <https://dx.doi.org/10.3402/ejpt.v6.27414> PubMed PMID: 25989952.

2. Acarturk C, Konuk E, Cetinkaya M, Senay I, Sijbrandij M, Gulen B, et al. The efficacy of eye movement desensitization and reprocessing for post-traumatic stress disorder and depression among Syrian refugees: results of a randomized controlled trial. Psychological Medicine. 2016;46(12):2583-93. doi: <https://dx.doi.org/10.1017/S0033291716001070> PubMed PMID: 27353367.

3. Adenauer H, Catani C, Gola H, Keil J, Ruf M, Schauer M, et al. Narrative exposure therapy for PTSD increases top-down processing of aversive stimuli--evidence from a randomized controlled treatment trial. BMC Neuroscience. 2011;12:127. doi: <https://dx.doi.org/10.1186/1471-2202-12-127> PubMed PMID: 22182346.

4. Ardani AR, Hosseini G, Bordbar MR, Talaei A, Toroghi HM. Effect of rivastigmine augmentation in treatment of male patients with combat-related chronic posttraumatic stress disorder a randomized controlled trial. Journal of Clinical Psychopharmacology (USA). 2017;37(1):54-60. doi: 10.1097/JCP.0000000000000624.

5. Azad Marzabadi E, Hashemi Zadeh SM. The effectiveness of mindfulness training in improving the quality of life of the war victims with post traumatic stress disorder (PTSD). Iranian Journal of Psychiatry. 2014;9(4):228-36. PubMed PMID: 25792991.

6. Bahadir-Yilmaz E, Oz F. The Effectiveness of Empowerment Program on Increasing Self-Esteem, Learned Resourcefulness, and Coping Ways in Women Exposed to Domestic Violence. Issues in Mental Health Nursing. 2018;39(2):135-41. doi: 10.1080/01612840.2017.1368750. PubMed PMID: WOS:000424943000005.

7. Bartzokis G, Lu PH, Turner J, Mintz J, Saunders CS. Adjunctive risperidone in the treatment of chronic combat-related posttraumatic stress disorder. Biological Psychiatry. 2005;57(5):474-9. doi: 10.1016/j.biopsych.2004.11.039. PubMed PMID: 15737661.

8. Bass J, Murray SM, Mohammed TA, Bunn M, Gorman W, Ahmed AM, et al. A Randomized Controlled Trial of a Trauma-Informed Support, Skills, and Psychoeducation Intervention for Survivors of Torture and Related Trauma in Kurdistan, Northern Iraq. Global health, science and practice. 2016;4(3):452-66. doi: <http://dx.doi.org/10.9745/GHSP-D-16-00017>. PubMed PMID: 619970238.

9. Becker ME, Hertzberg MA, Moore SD, Dennis MF, Beckham JC. A placebo-controlled trial of bupropion SR in the treatment of chronic posttraumatic stress disorder. Journal of Clinical Psychopharmacology (USA). 2007;27(2):193-7. doi: 10.1097/JCP.0b013e318032eaed.

10. Beidel DC, Frueh BC, Uhde TW, Wong N, Mentrikoski JM. Multicomponent behavioral treatment for chronic combat-related posttraumatic stress disorder: a randomized controlled trial. Journal of Anxiety Disorders. 2011;25(2):224-31. doi: <https://dx.doi.org/10.1016/j.janxdis.2010.09.006> PubMed PMID: 20951543; PubMed Central PMCID: PMCNIHMS238163.

11. Bichescu D, Neuner F, Schauer M, Elbert T. Narrative exposure therapy for political imprisonment-related chronic posttraumatic stress disorder and depression. Behaviour Research & Therapy. 2007;45(9):2212-20. doi: 10.1016/j.brat.2006.12.006. PubMed PMID: 17288990.

12. Bolton P, Bass JK, Zangana GA, Kamal T, Murray SM, Kaysen D, et al. A randomized controlled trial of mental health interventions for survivors of systematic violence in Kurdistan, Northern Iraq. BMC Psychiatry. 2014;14:360. doi: <https://dx.doi.org/10.1186/s12888-014-0360-2> PubMed PMID: 25551436.

13. Bonilla-Escobar FJ, Fandino-Losada A, Martinez-Buitrago DM, Santaella-Tenorio J, Tobon-Garcia D, Munoz-Morales EJ, et al. A randomized controlled trial of a transdiagnostic cognitive-behavioral intervention for Afro-descendants' survivors of systemic violence in Colombia. PLoS ONE [Electronic Resource]. 2018;13(12):e0208483. doi: <https://dx.doi.org/10.1371/journal.pone.0208483>. PubMed PMID: 30532155.

14. Bremner JD, Mishra S, Campanella C, Shah M, Kasher N, Evans S, et al. A pilot study of the effects of mindfulness-based stress reduction on post-traumatic stress disorder symptoms and brain response to traumatic reminders of combat in Operation Enduring Freedom/Operation Iraqi Freedom combat veterans with post-traumatic stress disorder. Frontiers in Psychiatry Vol 8 2017, ArtID 157. 2017;8. doi: 10.3389/fpsyt.2017.00157. PubMed PMID: 2017-38563-001.

15. Buhmann CB, Nordentoft M, Ekstroem M, Carlsson J, Mortensen EL. The effect of flexible cognitive-behavioural therapy and medical treatment, including antidepressants on post-traumatic stress disorder and depression in traumatised refugees: pragmatic randomised controlled clinical trial. British Journal of Psychiatry. 2016;208(3):252-9. doi: <https://dx.doi.org/10.1192/bjp.bp.114.150961> PubMed PMID: 26541687.

16. Carlson JG, Chemtob CM, Rusnak K, Hedlund NL, Muraoka MY. Eye movement desensitization and reprocessing (EMDR) treatment for combat-related posttraumatic stress disorder. Japanese Journal of Biofeedback Research. 1997;24:50-64. doi: 10.1023/A:1024448814268. PubMed PMID: 1999-13197-003.

17. Carlsson J, Sonne C, Vindbjerg E, Mortensen EL. Stress management versus cognitive restructuring in trauma-affected refugees-A pragmatic randomised study. Psychiatry Research. 2018;266:116-23. doi: <https://dx.doi.org/10.1016/j.psychres.2018.05.015>. PubMed PMID: 29859498.

18. Celik C, Ozdemir B, Ozmenler KN, Yelboga Z, Balikci A, Oznur T, et al. Efficacy of Paroxetine and Amitriptyline in Posttraumatic Stress Disorder: An Open-label Comparative Study. Klinik Psikofarmakoloji Bülteni-Bulletin of Clinical Psychopharmacology. 2011;21(3):179-85. doi: 10.5455/bcp.20110627111141.

19. Chard KM. An evaluation of cognitive processing therapy for the treatment of posttraumatic stress disorder related to childhood sexual abuse. Journal of Consulting & Clinical Psychology. 2005;73(5):965-71. doi: 10.1037/0022-006X.73.5.965. PubMed PMID: 16287396.

20. Chung MY, Min KH, Jun YJ, Kim SS, Kim WC, Jun EM. Efficacy and tolerability of mirtazapine and sertraline in Korean veterans with posttraumatic stress disorder: a randomized open label trial. Human Psychopharmacology. 2004;19(7):489-94. doi: 10.1002/hup.615. PubMed PMID: 15378676.

21. Classen C, Koopman C, Nevillmanning K, Spiegel D. A Preliminary Report Comparing Trauma-Focused and Present-Focused Group Therapy Against a Wait-Listed Condition Among Childhood Sexual Abuse Survivors with PTSD. Journal of Aggression, Maltreatment & Trauma. 2001;4(2):265-88. doi: 10.1300/J146v04n02_12.

22. Cloitre M, Stovall-McClough KC, Nooner K, Zorbas P, Cherry S, Jackson CL, et al. Treatment for PTSD related to childhood abuse: a randomized controlled trial. American Journal of Psychiatry. 2010;167(8):915-24. doi: <https://dx.doi.org/10.1176/appi.ajp.2010.09081247> PubMed PMID: 20595411.

23. Cook JM, Harb GC, Gehrman PR, Cary MS, Gamble GM, Forbes D, et al. Imagery rehearsal for posttraumatic nightmares: a randomized controlled trial. Journal of traumatic stress. 2010;23(5):553-63. doi: 10.1002/jts.20569

24. Davis LL, Jewell ME, Ambrose S, Farley J, English B, Bartolucci A, et al. A Placebo-Controlled Study of Nefazodone for the Treatment of Chronic Posttraumatic Stress Disorder: A Preliminary Study. Journal of Clinical Psychopharmacology. 2004;24(3). doi: 10.1097/01.jcp.0000125685.82219.1a.

25. Davis LL, Davidson JR, Ward LC, Bartolucci A, Bowden CL, Petty F. Divalproex in the treatment of posttraumatic stress disorder: a randomized, double-blind, placebo-controlled trial in a veteran population. Journal of Clinical Psychopharmacology. 2008;28(1):84-8. doi: <https://dx.doi.org/10.1097/JCP.0b013e318160f83b>. PubMed PMID: 18204347.

26. Devilly GJ, Spence SH, Rapee RM. Statistical and reliable change with eye movement desensitization and reprocessing: Treating trauma within a veteran population. Behavior Therapy. 1998;29(3):435-55. doi: <https://doi.org/10.1016/S0005-7894(98)80042-7>.

27. Edmond T, Rubin A, Wambach KG. The effectiveness of EMDR with adult female survivors of childhood sexual abuse. Social Work Research. 1999;23(2):103-16. doi: 10.1093/swr/23.2.103.

28. Engel CC, Litz B, Magruder KM, Harper E, Gore K, Stein N, et al. Delivery of self training and education for stressful situations (DESTRESS-PC): a randomized trial of nurse assisted online self-management for PTSD in primary care. General Hospital Psychiatry. 2015;37(4):323-8. doi: <https://dx.doi.org/10.1016/j.genhosppsych.2015.04.007> PubMed PMID: 25929985; PubMed Central PMCID: PMCNIHMS680713.

29. Feske U. Treating low-income and minority women with posttraumatic stress disorder: a pilot study comparing prolonged exposure and treatment as usual conducted by community therapists. Journal of Interpersonal Violence. 2008;23(8):1027-40. doi: <https://dx.doi.org/10.1177/0886260507313967>. PubMed PMID: 18292398.

30. Foa EB, McLean CP, Zang Y, Rosenfield D, Yadin E, Yarvis JS, et al. Effect of Prolonged Exposure Therapy Delivered Over 2 Weeks vs 8 Weeks vs Present-Centered Therapy on PTSD Symptom Severity in Military Personnel: A Randomized Clinical Trial. JAMA. 2018;319(4):354-64. doi: <https://dx.doi.org/10.1001/jama.2017.21242>. PubMed PMID: 29362795.

31. Ford JD, Grasso DJ, Greene CA, Slivinsky M, DeViva JC. Randomized clinical trial pilot study of prolonged exposure versus present centred affect regulation therapy for PTSD and anger problems with male military combat veterans. Clinical Psychology & Psychotherapy. 2018;25(5):641-9. doi: <https://dx.doi.org/10.1002/cpp.2194>. PubMed PMID: 29687524.

32. Franciskovic T, Sukovic Z, Janovic S, Stevanovic A, Nemcic-Moro I, Roncevic-Grzeta I, et al. Tianeptine in the combined treatment of combat related poasttraumatic stress disorder. Psychiatria Danubina. 2011;23(3):257-63. PubMed PMID: 2011-23671-010.

33. Franklin CL, Cuccurullo LA, Walton JL, Arseneau JR, Petersen NJ. Face to face but not in the same place: A pilot study of prolonged exposure therapy. Journal of Trauma and Dissociation. 2017;18(1):116-30. doi: <http://dx.doi.org/10.1080/15299732.2016.1205704> PubMed PMID: 611575198.

34. Friedman MJ, Marmar CR, Baker DG, Sikes CR, Farfel GM. Randomized, double-blind comparison of sertraline and placebo for posttraumatic stress disorder in a Department of Veterans Affairs setting. Journal of Clinical Psychiatry. 2007;68(5):711-20. doi: 10.4088/jcp.v68n0508. PubMed PMID: 17503980.

35. Galano MM, Grogan-Kaylor AC, Stein SF, Clark HM, Graham-Bermann SA. Posttraumatic stress disorder in Latina women: Examining the efficacy of the Moms' Empowerment Program. Psychological Trauma:Theory, Pesearch, Practice and Policy. 2017;9(3):344-51. doi: <https://dx.doi.org/10.1037/tra0000218>. PubMed PMID: 27869463.

36. Gamito P, Oliveira J, Rosa P, Morais D, Duarte N, OLiverira S, et al. PTSD Elderly War Veterans: A Clinical Controlled Pilot Study. Cyberpsychology, Behavior, and Social Networking. 2010;13(1):43-8. doi: 10.1089/cyber.2009.0237. PubMed PMID: 20528292.

37. Germain A, Richardson R, Moul DE, Mammen O, Haas G, Forman SD, et al. Placebo-controlled comparison of prazosin and cognitive-behavioral treatments for sleep disturbances in US Military Veterans. Journal of Psychosomatic Research. 2012;72(2):89-96. doi: <https://dx.doi.org/10.1016/j.jpsychores.2011.11.010> PubMed PMID: 22281448; PubMed Central PMCID: PMCNIHMS342239.

38. Hamner MB, Faldowski RA, Ulmer HG, Frueh BC, Huber MG, Arana GW. Adjunctive risperidone treatment in post-traumatic stress disorder: a preliminary controlled trial of effects on comorbid psychotic symptoms. International Clinical Psychopharmacology. 2003;18(1):1-8. doi: 10.1097/01.yic.0000050744.67514.6d. PubMed PMID: 12490768.

39. Hermenau K, Hecker T, Schaal S, Maedl A, Elbert T. Addressing post-traumatic stress and aggression by means of narrative exposure: A randomized controlled trial with ex-combatants in the eastern DRC. Journal of Aggression, Maltreatment and Trauma. 2013;22(8):916-34. doi: <http://dx.doi.org/10.1080/10926771.2013.824057> PubMed PMID: 369892869.

40. Hijazi AM, Lumley MA, Ziadni MS, Haddad L, Rapport LJ, Arnetz BB. Brief narrative exposure therapy for posttraumatic stress in Iraqi refugees: a preliminary randomized clinical trial. Journal of Traumatic Stress. 2014;27(3):314-22. doi: <https://dx.doi.org/10.1002/jts.21922> PubMed PMID: 24866253; PubMed Central PMCID: PMCNIHMS595425.

41. Himmerich H, Willmund GD, Zimmermann P, Wolf JE, Buhler AH, Kirkby KC, et al. Serum concentrations of TNF-alphas soluble receptors during psychotherapy in German soldiers suffering from combat-related PTSD. Psychiatria Danubina. 2016;28(3):293-8. doi: 10.4103/0366-6999.178039 PubMed PMID: 612523930.

42. Hinton DE, Pham T, Tran M, Safren SA, Otto MW, Pollack MH. CBT for Vietnamese refugees with treatment-resistant PTSD and panic attacks: a pilot study. Journal of Traumatic Stress. 2004;17(5):429-33. doi: 10.1023/B:JOTS.0000048956.03529.fa. PubMed PMID: 15633922; PubMed Central PMCID: PMCNIHMS144174.

43. Hinton DE, Chhean D, Pich V, Safren SA, Hofmann SG, Pollack MH. A randomized controlled trial of cognitive-behavior therapy for Cambodian refugees with treatment-resistant PTSD and panic attacks: a cross-over design. Journal of Traumatic Stress. 2005;18(6):617-29. doi: 10.1002/jts.20070. PubMed PMID: 16382423.

44. Jensen JA. An investigation of eye movement desensitization and reprocessing (EMD/R) as a treatment for posttraumatic stress disorder (PTSD) symptoms of Vietnam combat veterans. Behavior Therapy. 1994;25(2):311-25. doi: 10.1016/S0005-7894(05)80290-4. PubMed PMID: 1994-42215-001.

45. Jung K, Steil R. A randomized controlled trial on cognitive restructuring and imagery modification to reduce the feeling of being contaminated in adult survivors of childhood sexual abuse suffering from posttraumatic stress disorder. Psychotherapy & Psychosomatics. 2013;82(4):213-20. doi: <https://dx.doi.org/10.1159/000348450> PubMed PMID: 23712073.

46. Katz LS, Douglas S, Zaleski K, Williams J, Huffman C, Cojucar G. Comparing holographic reprocessing and prolonged exposure for women veterans with sexual trauma: A pilot randomized trial. Journal of Contemporary Psychotherapy. 2014;44(1):9-19. doi: <http://dx.doi.org/10.1007/s10879-013-9248-6> PubMed PMID: 52756441.

47. Keane TM, Fairbank JA, Caddell JM, Zimering RT. Implosive (flooding) therapy reduces symptoms of PTSD in Vietnam combat veterans. Behavior Therapy. 1989;20(2):245-60. doi: <https://doi.org/10.1016/S0005-7894(89)80072-3>.

48. Kearney DJ, McDermott K, Malte C, Martinez M, Simpson TL. Effects of participation in a mindfulness program for veterans with posttraumatic stress disorder: a randomized controlled pilot study. Journal of Clinical Psychology. 2013;69(1):14-27. doi: <https://dx.doi.org/10.1002/jclp.21911> PubMed PMID: 22930491.

49. Knaevelsrud C, Brand J, Lange A, Ruwaard J, Wagner B. Web-based psychotherapy for posttraumatic stress disorder in war-traumatized Arab patients: randomized controlled trial. Journal of Medical Internet Research. 2015;17(3):e71. doi: <https://dx.doi.org/10.2196/jmir.3582> PubMed PMID: 25799024.

50. Knaevelsrud C, Böttche M, Pietrzak RH, Freyberger HJ, Kuwert P. Efficacy and Feasibility of a Therapist-Guided Internet-Based Intervention for Older Persons with Childhood Traumatization: A Randomized Controlled Trial. American Journal of Geriatric Psychiatry. 2017;25(8):878-88. doi: 10.1016/j.jagp.2017.02.024. PubMed PMID: 124186984.

51. Kosten TR, Krystal JH, Giller EL, Frank J, Dan E. Alexithymia as a predictor of treatment response in post-traumatic stress disorder. Journal of Traumatic Stress. 1992;5(4):563-73. doi: 10.1016/j.biopsych.2013.05.017. PubMed PMID: 1993-18480-001.

52. Krupnick JL, Green BL, Stockton P, Miranda J, Krause E, Mete M. Group interpersonal psychotherapy for low-income women with posttraumatic stress disorder. Psychotherapy Research. 2008;18(5):497-507. doi: <https://dx.doi.org/10.1080/10503300802183678>. PubMed PMID: 18816001.

53. Krystal JH, Rosenheck RA, Cramer JA, Vessicchio JC, Jones KM, Vertrees JE, et al. Adjunctive risperidone treatment for antidepressant-resistant symptoms of chronic military service-related PTSD: a randomized trial. JAMA. 2011;306(5):493-502. doi: <https://dx.doi.org/10.1001/jama.2011.1080> PubMed PMID: 21813427.

54. Kubany ES, Hill EE, Owens JA. Cognitive trauma therapy for battered women with PTSD: preliminary findings. Journal of Traumatic Stress. 2003;16(1):81-91. doi: 10.1023/A:1022019629803. PubMed PMID: 12602656.

55. Kubany ES, Hill EE, Owens JA, Iannce-Spencer C, McCaig MA, Tremayne KJ, et al. Cognitive trauma therapy for battered women with PTSD (CTT-BW). Journal of Consulting & Clinical Psychology. 2004;72(1):3-18. doi: <https://dx.doi.org/10.1037/0022-006X.72.1.3> PubMed PMID: 14756610.

56. Lande RG, Williams LB, Francis JL, Gragnani C, Morin ML. Efficacy of biofeedback for post-traumatic stress disorder. Complementary Therapies in Medicine. 2010;18(6):256-9. doi: <https://dx.doi.org/10.1016/j.ctim.2010.08.004> PubMed PMID: 21130362.

57. Lau M, Kristensen E. Outcome of systemic and analytic group psychotherapy for adult women with history of intrafamilial childhood sexual abuse: a randomized controlled study. Acta Psychiatrica Scandinavica. 2007;116(2):96-104. doi: 10.1111/j.1600-0447.2006.00977.x. PubMed PMID: WOS:000248087900003.

58. Lindley SE, Carlson EB, Hill K. A randomized, double-blind, placebo-controlled trial of augmentation topiramate for chronic combat-related posttraumatic stress disorder. Journal of Clinical Psychopharmacology. 2007;27(6):677-81. doi: <https://dx.doi.org/10.1097/jcp.0b013e31815a43ee>. PubMed PMID: 18004136.

59. Lundqvist G, Svedin CG, Hansson K, Broman I. Group therapy for women sexually abused as children: mental health before and after group therapy. Journal of Interpersonal Violence. 2006;21(12):1665-77. doi: 10.1177/0886260506294986. PubMed PMID: 17065660.

60. Margolies SO, Rybarczyk B, Lynch J, Vrana S. Efficacy of a cognitive-behavioral treatment for insomnia among Afghanistan and Iraq (OEF/ OIF) veterans with PTSD. Sleep. 2011;34:A253-A4. doi: 0.1002/jclp.21970. PubMed PMID: 71510995.

61. McDonagh A, Friedman M, McHugo G, Ford J, Sengupta A, Mueser K, et al. Randomized trial of cognitive-behavioral therapy for chronic posttraumatic stress disorder in adult female survivors of childhood sexual abuse. Journal of Consulting & Clinical Psychology. 2005;73(3):515-24. doi: 10.1037/0022-006X.73.3.515. PubMed PMID: 15982149.

62. McLay RN, Wood DP, Webb-Murphy JA, Spira JL, Wiederhold MD, Pyne JM, et al. A randomized, controlled trial of virtual reality-graded exposure therapy for post-traumatic stress disorder in active duty service members with combat-related post-traumatic stress disorder. Cyberpsychology, behavior and social networking. 2011;14(4):223-9. doi: <https://dx.doi.org/10.1089/cyber.2011.0003> PubMed PMID: 21332375.

63. Meffert SM, Abdo AO, Alla OAA, Elmakki YOM, Omer AA, Yousif S, et al. A pilot randomized controlled trial of interpersonal psychotherapy for Sudanese refugees in Cairo, Egypt. Psychological Trauma: Theory, Research, Practice, and Policy. 2014;6(3):240-9. doi: 10.1037/a0023540.

64. Miyahira SD, Folen RA, Hoffman HG, Garcia-Palacios A, Spira JL, Kawasaki M. The effectiveness of VR exposure therapy for PTSD in returning warfighters. Annual Review of CyberTherapy and Telemedicine. 2012;10:128-32. doi: 10.3233/978-1-61499-121-2-128.

65. Monnelly EP, Ciraulo DA, Knapp C, Keane T. Low-Dose Risperidone as Adjunctive Therapy for Irritable Aggression in Posttraumatic Stress Disorder. Journal of Clinical Psychopharmacology. 2003;23(2). doi: 10.1097/00004714-200304000-00012.

66. Moradi AR, Moshirpanahi S, Parhon H, Mirzaei J, Dalgleish T, Jobson L. A pilot randomized controlled trial investigating the efficacy of MEmory Specificity Training in improving symptoms of posttraumatic stress disorder. Behaviour Research & Therapy. 2014;56:68-74. doi: <https://dx.doi.org/10.1016/j.brat.2014.03.002> PubMed PMID: 24705337.

67. Nakamura Y, Lipschitz DL, Donaldson GW, Kida Y, Williams SL, Landward R, et al. Investigating Clinical Benefits of a Novel Sleep-Focused Mind-Body Program on Gulf War Illness Symptoms: A Randomized Controlled Trial. Psychosomatic Medicine. 2017;79(6):706-18. doi: <https://dx.doi.org/10.1097/PSY.0000000000000469>. PubMed PMID: 28406803.

68. Narimani M, Sadeghieh Ahari S, Rajabi S. Comparison of efficacy of eye movement desensitization and reprocessing and cognitive behavioral therapy therapeutic methods for reducing anxiety and depression of Iranian combatant afflicted by post traumatic stress disorder. Journal of Applied Sciences. 2008;8(10):1932-7. doi: <http://dx.doi.org/10.3923/jas.2008.1932.1937>

69. Naylor JC, Dolber TR, Strauss JL, Kilts JD, Strauman TJ, Bradford DW, et al. A pilot randomized controlled trial with paroxetine for subthreshold PTSD in Operation Enduring Freedom/Operation Iraqi Freedom era veterans. Psychiatry Research. 2013;206(2-3):318-20. doi: 10.1016/j.psychres.2012.11.008. PubMed PMID: 2013-00092-001.

70. Neuner F, Onyut PL, Ertl V, Odenwald M, Schauer E, Elbert T. Treatment of posttraumatic stress disorder by trained lay counselors in an African refugee settlement: a randomized controlled trial. Journal of Consulting & Clinical Psychology. 2008;76(4):686-94. doi: <https://dx.doi.org/10.1037/0022-006X.76.4.686> PubMed PMID: 18665696.

71. Neuner F, Schauer M, Klaschik C, Karunakara U, Elbert T. A comparison of narrative exposure therapy, supportive counseling, and psychoeducation for treating posttraumatic stress disorder in an African refugee settlement. Journal of Consulting & Clinical Psychology. 2004;72(4):579-87. doi: 10.1037/0022-006X.72.4.579. PubMed PMID: 15301642.

72. Niles BL, Klunk-Gillis J, Ryngala DJ, Silberbogen AK, Paysnick A, EJ. W. Comparing mindfulness and psychoeducation treatments for combat-related PTSD using a telehealth approach. Psychological Trauma: Theory, Research, Practice, and Policy. 2012;4:538-47. doi: 10.1037/a0026161.

73. Otto MW, Hinton D, Korbly NB, Chea A, Ba P, Gershuny BS, et al. Treatment of pharmacotherapy-refractory posttraumatic stress disorder among Cambodian refugees: a pilot study of combination treatment with cognitive-behavior therapy vs sertraline alone. Behav Res Ther. 2003;41(11):1271-6. doi: 10.1016/s0005-7967(03)00032-9. PubMed PMID: 14527527.

74. Owens GP, Pike JL, Chard KM. Treatment effects of cognitive processing therapy on cognitive distortions of female child sexual abuse survivors. Behavior Therapy. 2001;32(3):413-24. doi: <https://doi.org/10.1016/S0005-7894(01)80028-9>. PubMed PMID: 2002-12369-001.

75. Panahi Y, Moghaddam BR, Sahebkar A, Nazari MA, Beiraghdar F, Karami G, et al. A randomized, double-blind, placebo-controlled trial on the efficacy and tolerability of sertraline in Iranian veterans with post-traumatic stress disorder. Psychological Medicine. 2011;41(10):2159-66. doi: <https://dx.doi.org/10.1017/S0033291711000201> PubMed PMID: 21349225.

76. Paunovic N, Ost LG. Cognitive-behavior therapy vs exposure therapy in the treatment of PTSD in refugees. Behav Res Ther. 2001;39(10):1183-97. doi: 10.1016/S0005-7967(00)00093-0. PubMed PMID: 11579988.

77. Polusny MA, Erbes CR, Thuras P, Moran A, Lamberty GJ, Collins RC, et al. Mindfulness-based stress reduction for posttraumatic stress disorder among veterans: a randomized clinical trial. JAMA. 2015;314(5):456-65. doi: <https://dx.doi.org/10.1001/jama.2015.8361> PubMed PMID: 26241597.

78. Possemato K, Bergen-Cico D, Treatman S, Allen C, Wade M, Pigeon W. A randomized clinical trial of primary care brief mindfulness training for veterans with PTSD. Journal of Clinical Psychology. 2016;72(3):179-93. doi: <https://dx.doi.org/10.1002/jclp.22241> PubMed PMID: 26613203.

79. Raskind MA, Peskind ER, Hoff DJ, Hart KL, Holmes HA, Warren D, et al. A parallel group placebo controlled study of prazosin for trauma nightmares and sleep disturbance in combat veterans with post-traumatic stress disorder. Biological Psychiatry. 2007;61(8):928-34. doi: <https://dx.doi.org/10.1016/j.biopsych.2006.06.032>. PubMed PMID: 17069768.

80. Raskind MA, Peterson K, Williams T, Hoff DJ, Peskind ER. A trial of prazosin for combat trauma PTSD with nightmares in active-duty soldiers returned from Iraq and Afghanistan. American Journal of Psychiatry. 2013;170(9):1003-10. doi: 10.1176/appi.ajp.2013.12081133.

81. Rauch SA, Kim H, Powell C, Tuerk PW, Simon NM, Acierno R, et al. Efficacy of prolonged exposure therapy, sertraline hydrochloride, and their combination among combat veterans with posttraumatic stress disorder: A randomized clinical trial. JAMA Psychiatry. 2019;76(2):117-25. doi: <http://dx.doi.org/10.1001/jamapsychiatry.2018.3412>. PubMed PMID: 2019-07144-001.

82. Ready DJ, Gerardi RJ, Backscheider AG, Mascaro N, Rothbaum BO. Comparing virtual reality exposure therapy to present-centered therapy with 11 U.S. Vietnam veterans with PTSD. Cyberpsychol Behav Soc Netw. 2010;13(1):49-54. doi: 10.1089/cyber.2009.0239. PubMed PMID: 20528293.

83. Reed GL, Enright RD. The effects of forgiveness therapy on depression, anxiety, and posttraumatic stress for women after spousal emotional abuse. Journal of Consulting & Clinical Psychology. 2006;74(5):920-9. doi: <https://dx.doi.org/10.1037/0022-006X.74.5.920>. PubMed PMID: 17032096.

84. Reger GM, Koenen-Woods P, Zetocha K, Smolenski DJ, Holloway KM, Rothbaum BO, et al. Randomized controlled trial of prolonged exposure using imaginal exposure vs. virtual reality exposure in active duty soldiers with deployment-related posttraumatic stress disorder (PTSD). Journal of Consulting and Clinical Psychology. 2016;84(11):946-59. doi: <http://dx.doi.org/10.1037/ccp0000134> PubMed PMID: 612057864.

85. Reich DB, Winternitz S, Hennen J, Watts T, Stanculescu C. A preliminary study of risperidone in the treatment of posttraumatic stress disorder related to childhood abuse in women. Journal of Clinical Psychiatry. 2004;65(12):1601-6. doi: 0.4088/jcp.v65n1204. PubMed PMID: 15641864.

86. Resick PA, Wachen JS, Mintz J, Young-McCaughan S, Roache JD, Borah AM, et al. A randomized clinical trial of group cognitive processing therapy compared with group present-centered therapy for PTSD among active duty military personnel. Journal of Consulting & Clinical Psychology. 2015;83(6):1058-68. doi: <https://dx.doi.org/10.1037/ccp0000016> PubMed PMID: 25939018.

87. Rogers S, Silver SM, Goss J, Obenchain J, Willis A, Whitney RL. A Single Session, Group Study of Exposure and Eye Movement Desensitization and Reprocessing in Treating Posttraumatic Stress Disorder Among Vietnam War Veterans: Preliminary Data. J Anxiety Disord. 1999;13(1):119-30. doi: <https://doi.org/10.1016/S0887-6185(98)00043-7>.

88. Shapiro E, Laub B, Rosenblat O. Early EMDR intervention following intense rocket attacks on a town: A randomised clinical trial. Clinical Neuropsychiatry: Journal of Treatment Evaluation. 2018;15(3):194-205. doi: 10.1080/20008198.2019.1695486. PubMed PMID: 2018-27261-003.

89. Sikkema KJ, Hansen NB, Kochman A, Tarakeshwar N, Neufeld S, Meade CS, et al. Outcomes from a group intervention for coping with HIV/AIDS and childhood sexual abuse: reductions in traumatic stress. AIDS & Behavior. 2007;11(1):49-60. doi: <https://dx.doi.org/10.1007/s10461-006-9149-8>. PubMed PMID: 16858634.

90. Sikkema KJ, Ranby KW, Meade CS, Hansen NB, Wilson PA, Kochman A. Reductions in traumatic stress following a coping intervention were mediated by decreases in avoidant coping for people living with HIV/AIDS and childhood sexual abuse. Journal of Consulting & Clinical Psychology. 2013;81(2):274-83. doi: <https://dx.doi.org/10.1037/a0030144> PubMed PMID: 23025248; PubMed Central PMCID: PMCNIHMS433358.

91. Smajkic A, Weine S, Djuric-Bijedic Z, Boskailo E, Lewis J, Pavkovic I. Sertraline, paroxetine, and venlafaxine in refugee posttraumatic stress disorder with depression symptoms. Journal of Traumatic Stress. 2001;14(3):445-52. doi: <https://dx.doi.org/10.1023/A:1011177420069>. PubMed PMID: 11534876.

92. Sonne C, Carlsson J, Bech P, Elklit A, Mortensen EL. Treatment of trauma-affected refugees with venlafaxine versus sertraline combined with psychotherapy-A randomised study. BMC Psychiatry Vol 16 2016, ArtID 383. 2016;16. doi: 10.1186/s12888-016-1081-5. PubMed PMID: 2016-54614-001.

93. Spidel A, Lecomte T, Kealy D, Daigneault I. Acceptance and commitment therapy for psychosis and trauma: Improvement in psychiatric symptoms, emotion regulation, and treatment compliance following a brief group intervention. Psychology and Psychotherapy-Theory Research and Practice. 2018;91(2):248-61. doi: 10.1111/papt.12159. PubMed PMID: WOS:000434168200007.

94. Stein MB, Kline NA, Matloff JL. Adjunctive olanzapine for SSRI-resistant combat-related PTSD: A double-blind, placebo-controlled study. The American Journal of Psychiatry. 2002;159(10):1777-9. doi: 10.1176/appi.ajp.159.10.1777. PubMed PMID: 2002-04602-025.

95. Stenmark H, Catani C, Neuner F, Elbert T, Holen A. Treating PTSD in refugees and asylum seekers within the general health care system. A randomized controlled multicenter study. Behaviour Research & Therapy. 2013;51(10):641-7. doi: <https://dx.doi.org/10.1016/j.brat.2013.07.002> PubMed PMID: 23916633.

96. Teng EJ, Bailey SD, Chaison AD, Petersen NJ, Hamilton JD, Dunn NJ. Treating comorbid panic disorder in veterans with posttraumatic stress disorder. Journal of Consulting & Clinical Psychology. 2008;76(4):704-10. doi: <https://dx.doi.org/10.1037/0022-006X.76.4.710> PubMed PMID: 18665698.

97. Ter Heide FJ, Mooren TM, Kleijn W, de Jongh A, Kleber RJ. EMDR versus stabilisation in traumatised asylum seekers and refugees: results of a pilot study. European Journal of Psychotraumatology. 2011;2. doi: <https://dx.doi.org/10.3402/ejpt.v2i0.5881> PubMed PMID: 22893808.

98. Ter Heide FJ, Mooren TM, van de Schoot R, de Jongh A, Kleber RJ. Eye movement desensitisation and reprocessing therapy v. stabilisation as usual for refugees: randomised controlled trial. British Journal of Psychiatry. 2016;209(4):311-8. doi: <https://dx.doi.org/10.1192/bjp.bp.115.167775> PubMed PMID: 26892849.

99. Thorp SR, Glassman LH, Wells SY, Walter KH, Gebhardt H, Twamley E, et al. A randomized controlled trial of prolonged exposure therapy versus relaxation training for older veterans with military-related PTSD. Journal of Anxiety Disorders. 2019;64:45-54. doi: <http://dx.doi.org/10.1016/j.janxdis.2019.02.003>. PubMed PMID: 2019-27063-007.

100. Ulmer CS, Edinger JD, Calhoun PS. A multi-component cognitive-behavioral intervention for sleep disturbance in veterans with PTSD: a pilot study. J Clin Sleep Med. 2011;7(1):57-68. doi: 10.5664/jcsm.28042 PubMed PMID: 21344046.

101. Van der Kolk BA, Dreyfuss D, Michaels M, Shera D, Berkowitz R, Fisler R, et al. Fluoxetine in posttraumatic stress disorder. Journal of Clinical Psychiatry. 1994;55(12):517-22. PubMed PMID: 25026437.

102. Wahbeh H, Goodrich E, Goy E, Oken BS. Mechanistic pathways of mindfulness meditation in combat veterans with posttraumatic stress disorder. Journal of Clinical Psychology. 2016;72(4):365-83. doi: <https://dx.doi.org/10.1002/jclp.22255> PubMed PMID: 26797725; PubMed Central PMCID: PMCNIHMS744049 [Available on 04/01/17].

103. Wang SJ, Bytyci A, Izeti S, Kallaba M, Rushiti F, Montgomery E, et al. A novel bio-psycho-social approach for rehabilitation of traumatized victims of torture and war in the post-conflict context: a pilot randomized controlled trial in Kosovo. Conflict & Health [Electronic Resource]. 2016;10:34. doi: <https://dx.doi.org/10.1186/s13031-016-0100-y> PubMed PMID: 28191034.

104. Weiss WM, Murray LK, Zangana GA, Mahmooth Z, Kaysen D, Dorsey S, et al. Community-based mental health treatments for survivors of torture and militant attacks in Southern Iraq: a randomized control trial. BMC Psychiatry. 2015;15:249. doi: <https://dx.doi.org/10.1186/s12888-015-0622-7> PubMed PMID: 26467303.

105. Yeomans PD, Forman EM, Herbert JD, Yuen EK. A randomized trial of a reconciliation workshop with and without PTSD psychoeducation in Burundian sample. Journal of Traumatic Stress. 2010;23(3):305-12. doi: <http://dx.doi.org/10.1002/jts.20531>

106. Yurtsever A, Konuk E, Akyuz T, Zat Z, Tukel F, Cetinkaya M, et al. An Eye Movement Desensitization and Reprocessing (EMDR) Group Intervention for Syrian Refugees With Post-traumatic Stress Symptoms: Results of a Randomized Controlled Trial. Frontiers in Psychology. 2018;9:493. doi: <https://dx.doi.org/10.3389/fpsyg.2018.00493>. PubMed PMID: 29946275.

107. Zlotnick C, Shea TM, Rosen K, Simpson E, Mulrenin K, Begin A, et al. An affect-management group for women with posttraumatic stress disorder and histories of childhood sexual abuse. Journal of Traumatic Stress. 1997;10(3):425-36. doi: 10.1023/a:1024841321156. PubMed PMID: 9246650.

108. Zohar J, Amital D, Miodownik C, Kotler M, Bleich A, Lane RM, et al. Double-blind placebo-controlled pilot study of sertraline in military veterans with posttraumatic stress disorder. Journal of Clinical Psychopharmacology. 2002;22(2):190-5. doi: 10.1097/00004714-200204000-00013. PubMed PMID: 11910265.
