# Supplementary material for: Psychological and pharmacological interventions for posttraumatic stress disorder and comorbid mental health problems following complex traumatic events: Systematic review and component network meta-analysis
Source: PLoS Med. 2020 Aug 19;17(8):e1003262. doi: 10.1371/journal.pmed.1003262 (PMC7446790; doi:10.1371/journal.pmed.1003262)
Supplement: S3 Table — -, significant sources of bias; +, potential sources of bias; ++, minimal sources of bias; NA, not applicable; NR, not reported. (DOCX) [file pmed.1003262.s005.docx]

S3 Table Risk of bias assessments for non-randomised controlled trials

|  | **Population bias** | | | **Allocation** | | | | | | | **Outcomes** | | **Analyses** | | | **Summary** | |
| --- | --- | --- | --- | --- | --- | --- | --- | --- | --- | --- | --- | --- | --- | --- | --- | --- | --- |
| **First Author (Year)** | **1.1 Is the source population or source area well described?** | **1.2 Is the eligible population or area representative of the source population or area?** | **1.3 Do the selected participants or areas represent the eligible population or area?** | **2.1 Allocation to intervention (or comparison). How was selection bias minimised?** | **2.2 Were interventions (and comparisons) well described and appropriate?** | **2.4 Were participants or investigators blind to exposure and comparison?** | **2.6 Was contamination acceptably low?** | **2.7 Were other interventions similar in both groups?** | **2.8 Were all participants accounted for at study conclusion?** | **2.10 Did the intervention or control comparison reflect usual UK practice?** | **3.1 Were outcome measures reliable?** | **3.3 Were all important outcomes assessed?** | **4.1 Were exposure and comparison groups similar at baseline? If not, were these adjusted?** | **4.2 Was intention to treat (ITT) analysis conducted?** | **4.4 Were the estimates of effect size given or calculable?** | **5.1 Are the study results internally valid (i.e. unbiased)?** | **5.2 Are the findings generalisable to the source population (i.e. externally valid)?** |
| King et al [1] | ++ | ++ | + | + | ++ | - | ++ | ++ | ++ | NA | + | + | ++ | + | ++ | + | + |
| Levi et al [2] | ++ | ++ | + | - | ++ | - | NR | NR | + | NA | + | + | ++ | + | ++ | - | + |
| Morgan et al [3] | + | + | + | + |  | - | ++ | NR | ++ | NA | + | - | + | + | + | + | + |
| Saxe et al [4] | + | + | ++ | + | + | - | ++ | - | + | NA | + | - | ++ | - | ++ | - | + |
| Pivac et al [5] | + | + | NR | NR | + | - | NR | NR | NR | NA | + | + | + | NR | - | NR | + |
| Lundqvist et al [6] | + | + | + | - | - | - | NR | - | + | NA | + | - | + | - | + | - | + |
| Salo et al [7] | ++ | + | ++ | - | + | NR | NR | NR | - | ++ | ++ | ++ | - | - | ++ | + | ++ |
| Narimani et al [8] | - | + | ++ | + | ++ | NR | NR | NR | - | ++ | + | + | NR | NR | - | + | - |
| Kruse et al [9] | ++ | + | ++ | + | ++ | NR | NR | + | - | ++ | ++ | + | ++ | - | ++ | + | ++ |

- significant sources of bias; + potential sources of bias; ++ minimal sources of bias; NA = not applicable; NR = not reported.

References

1. King AP, Erickson TM, Giardino ND, Favorite T, Rauch SA, Robinson E, et al. A pilot study of group mindfulness-based cognitive therapy (MBCT) for combat veterans with posttraumatic stress disorder (PTSD). Depress Anxiety. 2013;30(7):638-45. doi: <https://dx.doi.org/10.1002/da.22104> PubMed PMID: 23596092; PubMed Central PMCID: PMCNIHMS671148.

2. Levi O, Bar-Haim Y, Kreiss Y, Fruchter E. Cognitive-behavioural therapy and psychodynamic psychotherapy in the treatment of combat-related post-traumatic stress disorder: a comparative effectiveness study. Clin Psychol Psychother. 2016;23(4):298-307. doi: 10.1002/cpp.1969 PubMed PMID: 117169055.

3. Morgan T, Cummings AL. Change experienced during group therapy by female survivors of childhood sexual abuse. Journal of Consulting and Clinical Psychology. 1999;67(1):28-36. doi: 10.1037/0022-006X.67.1.28.

4. Saxe BJ, Johnson SM. An empirical investigation of group treatment for a clinical population of adult female incest survivors. Journal of Child Sexual Abuse. 1999;8(1):67-88. doi: <https://doi.org/10.1300/J070v08n01_05>. PubMed PMID: 107099962.

5. Pivac N, Kozaric-Kovacic D, Muck-Seler D. Olanzapine versus fluphenazine in an open trial in patients with psychotic combat-related post-traumatic stress disorder. Psychopharmacology (Berl). 2004;175(4):451-6. doi: 10.1007/s00213-004-1849-z. PubMed PMID: 2004-19344-008.

6. Lundqvist G, Svedin CG, Hansson K, Broman I. Group therapy for women sexually abused as children: mental health before and after group therapy. J Interpers Violence. 2006;21(12):1665-77. doi: 10.1177/0886260506294986. PubMed PMID: 17065660.

7. Salo J, Punamaki R-L, Qouta S, El Sarraj E. Individual and group treatment and self and other representations predicting posttraumatic recovery among former political prisoners. Traumatology. 2008;14(2):45-61. doi: 10.1177/1534765608319079. PubMed PMID: 2009-10421-005.

8. Narimani M, Sadeghieh Ahari S, Rajabi S. Comparison of efficacy of eye movement desensitization and reprocessing and cognitive behavioral therapy therapeutic methods for reducing anxiety and depression of Iranian combatant afflicted by post traumatic stress disorder. J Appl Sci. 2008;8(10):1932-7. doi: <http://dx.doi.org/10.3923/jas.2008.1932.1937>

9. Kruse J, Joksimovic L, Cavka M, Woller W, Schmitz N. Effects of trauma-focused psychotherapy upon war refugees. J Trauma Stress. 2009;22(6):585-92. doi: <https://dx.doi.org/10.1002/jts.20477> PubMed PMID: 19960519.
