# Supplementary material for: Psychological and pharmacological interventions for posttraumatic stress disorder and comorbid mental health problems following complex traumatic events: Systematic review and component network meta-analysis
Source: PLoS Med. 2020 Aug 19;17(8):e1003262. doi: 10.1371/journal.pmed.1003262 (PMC7446790; doi:10.1371/journal.pmed.1003262)
Supplement: S4 Table — BDI, Beck depression inventory; CAPS, clinician-administered PTSD scale; CBT, cognitive behavioural therapy; EMDR, eye movement desensitisation and reprocessing therapy; IPT, interpersonal therapy; PANSS, positive and negative syndrome scale; PTSD, posttraumatic stress disorder; SSRI, selective serotonin reuptake inhibitor; TF-CBT, trauma-focused cognitive behavioural therapy. (DOCX) [file pmed.1003262.s006.docx]

S4 Table Effect sizes (standardised mean difference) for psychological and pharmacological interventions versus control in all populations

|  | Post-treatment | Number of trials | Number of participants | <6 month follow-up | Number of trials | Number of participants |
| --- | --- | --- | --- | --- | --- | --- |
| **PTSD Symptoms** | | | | | | |
| All psychological interventions | -0.82 (-1.02 to 0.63) I^2^=83.5%, p=0.000  Equivalent to CAPS total: -16.45 (-20.46 to 12.64) | 46 | 3389 | -0.45 (-0.82 to-0.08)  I^2^=79.4%, p=<.001 | 10 | 738 |
| TF-CBT | -1.08 (-1.40 to 0.76) I^2^=85.3%, p=0.000  Equivalent to CAPS total: -21.66 (-28.08 to 15.24) | 22 | 1542 | -0.64 (-1.10 to -0.18) I^2^=44.9%, p=0.14 | 4 | 206 |
| EMDR | -0.99 (-1.44 to -0.54) I^2^=70.4%, p=0.000  Equivalent to CAPS total: -19.86 (-28.88 to -10.83) | 9 | 315 |  |  |  |
| IPT | -1.41 (-1.97 to -0.85) I^2^=0%, p=0.826 Equivalent to CAPS total: -28.28 (-39.52 to -17.05) | 2 | 66 |  |  |  |
| Mindfulness | -0.26 (-0.55 to 0.04) I^2^=0%, p=0.517  Equivalent to CAPS total: -5.22 (-11.03 to 0.80) | 3 | 183 | -0.08 (-0.68 to 0.52) I^2^=59%, p=0.12 | 2 | 109 |
| Non trauma-focused CBT | -0.05 (-0.23 to 0.13) I^2^=0%, p=0.566  Equivalent to CAPS total: -1.00 (-4.61 to 2.61) | 3 | 548 | -0.02 (-0.25 to 0.20) I^2^=0%, p=0.51 | 2 | 305 |
| Phase based | -1.13 (-1.54 to -0.73) I^2^=35.9%, p=0.168  Equivalent to CAPS total: -22.67 (-30.89 to -14.64 | 6 | 190 |  |  |  |
| Tricyclic antidepressants and monoamine oxidase inhibitors | -0.50  (-1.22 to 0.22) I^2^=87%, p<0.001  Equivalent to CAPS total:-10.03 (-24.47 to 4.41) | 7 | 338 |  |  |  |
| SSRIs | -0.60  (-1.64 to 0.41) I^2^=92.1%, p<0.001  Equivalent to CAPS total: -12.04 (-32.90 to 8.22) | 4 | 293 |  |  |  |
| Anti-psychotics | -0.45  (-0.85 to -0.05) I^2^=51.2%, p=0.14  Equivalent to CAPS total: -9.03 (-17.05 to -1.00) | 5 | 365 |  |  |  |
| Anti-convulsants | -0.16  (-0.77 to 0.45) I^2^=45.5%, p=0.18  Equivalent to CAPS total: -3.21 (-15.45 to 9.03) | 2 | 106 |  |  |  |
| Prazosin | -0.52  (-1.03 to -0.02) I^2^=41.4%, p=0.18  Equivalent to CAPS total: -10.43 (-20.67 to -0.40) | 3 | 110 |  |  |  |
| **Depression** | | | | | | |
| All psychological interventions | -0.87 (-1.11 to -0.63) I^2^=82.7%, p=0.00074  Equivalent to BDI: -8.53 (-10.88 to -6.18) | 31 | 2075 | -0.51  (-0.80, -0.22) I^2^=48%, p=0.05 | 5 | 410 |
| TF-CBT | -0.91 (-1.31 to -0.51) I^2^=87.3%, p0.000  Equivalent to BDI: -8.92 (-12.84 to -5.00) | 14 | 1042 | -0.72 (-1.43 to -0.01) I^2^=56.6%, p=0.12 | 3 | 104 |
| EMDR | -0.85  (-1.43 to -0.26) I^2^=78%, p=0.022  Equivalent to BDI: -8.33 (-14.02 to -2.55) | 7 | 253 |  |  |  |
| IPT | -1.17 (-1.71 to -0.62) I^2^=0%, p=0.524  Equivalent to BDI: -11.47 (-16.77 to -6.08) | 2 | 66 |  |  |  |
| Mindfulness | -0.43 (-0.73 to -0.13) I^2^=0%, p=0.685  Equivalent to BDI: -4.22 (-7.16 to -1.27) | 3 | 186 | -0.08 (-0.68 to 0.52) I^2^=59%, p=0.12 | 2 | 109 |
| Non trauma-focused CBT | -0.05 (-0.48, 0.39), I^2^=10%, p=0.292  Equivalent to BDI: -0.49 (-4.71 to 3.82) | 2 | 93 |  |  |  |
| Phase based | -1.03 (-1.43 to -0.63) I^2^=10.9%, p=0.339  Equivalent to BDI: -10.10  (-14.02 to -6.18) | 4 | 133 |  |  |  |
| Tricyclic antidepressants and monoamine oxidase inhibitors | 0.07  (-0.20 to 0.34) I^2^=0%, p=0.63  Equivalent to BDI: 0.69 (-1.96 to 3.33) | 3 | 220 |  |  |  |
| Anti-psychotics | -0.71 (-1.44 to 0.03) I^2^=58.3%, p=0.12  Equivalent to BDI: -6.96 (-14.12 to 0.29) | 2 | 266 |  |  |  |
| Anti-convulsants | 0.02 (-0.37 to 0.40) I^2^=0%, p=0.40  Equivalent to BDI: 0.20 (-3.63 to 3.92) | 2 | 106 |  |  |  |
| Prazosin | -0.37 (-1.21 to 0.47) I^2^=68.5%, p=0.08  Equivalent to BDI: -3.63 (-11.86 to 4.61) | 2 | 76 |  |  |  |
| **Anxiety** | | | | | | |
| All psychological interventions | -1.03  (-1.44 to -0.61) I^2^=90%, p=0.000 | 15 | 1395 |  |  |  |
| TF-CBT | -0.60 (-1.01 to -0.19) I^2^=85.5%, p=0.000 | 8 | 832 |  |  |  |
| EMDR | -1.05 (-1.50 to -0.61) I^2^=9.2%, p=0.347 | 4 | 102 |  |  |  |
| **Quality of life** | | | | | | |
| All psychological interventions | 0.33 (-0.01 to 0.66) I^2^=57.3%, p=0.021 | 6 | 401 |  |  |  |
| TF-CBT | 0.23 (-0.33 to 0.79) I^2^=73.9%, p=0.009 | 4 | 260 |  |  |  |
| **Sleep quality** | | | | | | |
| All psychological interventions | -1.00 (-1.49 to-0.51) I^2^=28.8%, p=0.245 | 3 | 111 |  |  |  |
| TF-CBT | -1.30 (-1.87 to -0.73) I^2^=0%, p=0.747 | 2 | 59 |  |  |  |
| Prazosin | -0.73 (-1.12 to -0.34) I^2^=0%, p=0.486 | 3 | 109 |  |  |  |
| **Positive and negative affect** | | | | | | |
| Anti-psychotics  PANSS-positive | -1.75 (-4.05 to 0.54) I^2^=76.9%, p=0.01 | 3 | 329 |  |  |  |
| Anti-psychotics  PANSS-negative | 0.54 (-0.14 to 1.22) I^2^=0%, p=0.66 | 2 | 284 |  |  |  |
| Anti-psychotics  PANSS-total | 0.04 (-2.08 to 2.16) I^2^=0%, p=0.43 | 2 | 284 |  |  |  |
| **Emotional dysregulation** | | | | | | |
| All psychological interventions | -0.38 (-0.88 to 0.12) I^2^=74.5%, p=0.001 | 7 | 289 | -0.42 (-1.53 to 0.69) I^2^=72.3%, p=0.6 | 2 | 51 |
| TF-CBT | -0.17 (-0.82 to 0.49) I^2^=60.6%, p=0.079 | 3 | 103 | -0.42 (-1.53 to 0.69) I^2^=72.3%, p=0.6 | 2 | 51 |
| Phase-based | -0.76 (-1.79, 0.27), I^2^=83.4%, p=0.002 | 3 | 118 |  |  |  |
| **Interpersonal problems** | | | | | | |
| All psychological interventions | -0.59 (-1.28 to 0.11) I^2^=61.9%, p=0.105 | 2 | 94 |  |  |  |
| Phase-based | -0.59 (-1.28 to 0.11) I^2^=61.9%, p=0.015 | 2 | 94 |  |  |  |
| **Negative self-concept** | | | | | | |
| All psychological interventions | 1.81 (0.73 to 2.89) I^2^=90%, p=0.000 | 5 | 215 |  |  |  |
| TF-CBT | 2.22 (0.75 to 3.70) I^2^=90.4%, p=0.000 | 3 | 145 |  |  |  |

BDI: Beck depression inventory, CAPS: clinician administered PTSD scale, CBT – cognitive behavioural therapy, EMDR – eye movement desensitisation and reprocessing therapy, IPT – interpersonal therapy, PANSS – positive and negative syndrome scale, PTSD – post-traumatic stress disorder, SSRI – selective serotonin reuptake inhibitor, TF-CBT – trauma-focused cognitive behavioural therapy.
