# Supplementary material for: Psychological and pharmacological interventions for posttraumatic stress disorder and comorbid mental health problems following complex traumatic events: Systematic review and component network meta-analysis
Source: PLoS Med. 2020 Aug 19;17(8):e1003262. doi: 10.1371/journal.pmed.1003262 (PMC7446790; doi:10.1371/journal.pmed.1003262)
Supplement: S5 Table — EMDR, eye movement desensitisation and reprocessing therapy; PTSD, posttraumatic stress disorder; TF-CBT, trauma-focused cognitive behavioural therapy. (DOCX) [file pmed.1003262.s007.docx]

S5 Table Effect sizes (standardised mean difference) versus for psychological interventions versus control for complex trauma exposure sub-groups

|  |  | Post-treatment | Number of trials | Number of participants | <6 month follow-up | Number of trials | Number of participants |
| --- | --- | --- | --- | --- | --- | --- | --- |
| **Veterans** | **PTSD symptoms** | | | | | | |
|  | All psychological interventions | -0.54 (-0.78 to -0.30) I^2^=53%, p=0.051 | 15 | 868 | -0.20 (-0.72 to 0.33) I^2^=63.1%, p=0.04 | 4 | 180 |
|  | TF-CBT | -0.80 (-1.14 to -0.45) I^2^=43.1%, p=0.108 | 7 | 458 |  |  |  |
|  | EMDR | -0.58 (-1.00 to -0.16) I^2^=11.1%, p=0.337 | 4 | 106 |  |  |  |
|  | Mindfulness | -0.26 (-0.55 to 0.04) I^2^=0%, p=0.517 | 3 | 186 | -0.08 (-0.68 to 0.52) I^2^=59%, p=0.12 | 2 | 109 |
|  | **Depression** | | | | | | |
|  | All psychological interventions | -0.56 (-0.84 to -0.28) I^2^=46.8%, p=0.061 | 11 | 445 | -0.38 (-0.78 to 0.01) I^2^=42.3%, p=0.14 | 2 | 90 |
|  | TF-CBT | -1.02 (-1.72 to -0.32) I^2^=51%, p=0.130 | 3 | 112 |  |  |  |
|  | EMDR | -0.91  (2.28 to -0.47) I^2^=77.7%, p=0.034 | 2 | 44 |  |  |  |
|  | Mindfulness | -0.43 (-0.7 to -0.13) I^2^=0%, p=0.685 | 3 | 186 | -0.41 (-0.79 to -0.02) I^2^=0%, p=0.44 | 2 | 109 |
|  | **Anxiety** | | | | | | |
|  | EMDR | -0.89 (-1.45 to -0.33) I^2^=10.6%, p=0.33 | 2 | 44 |  |  |  |
| **Childhood sexual abuse** | **PTSD symptoms** | | | | | | |
|  | All psychological interventions | -0.83 (-1.30 to -0.35) I^2^=88.5%, p=0.000 | 10 | 737 | -0.27 (-0.71 to 0.17) I^2^=53.6%, p=0.69 | 3 | 323 |
|  | TF-CBT | -1.22 (-2.40 to -0.05) I^2^=90.3%, p=0.000 | 3 | 153 |  |  |  |
|  | Non trauma-focused CBT | 0.01 (-0.20 to 0.21) I^2^=0%, p=0.587 | 2 | 368 |  |  |  |
|  | **Depression** | | | | | | |
|  | All psychological interventions | -0.82 (-1.10 to -0.55) I^2^=0%, p=0.627 | 5 | 234 | -0.52 (-0.99 to -0.04) I^2^=0%, p=0.69 | 2 | 76 |
|  | TF-CBT | -0.50 (-0.95, -0.05) I^2^=0%, p=0.627 | 2 | 79 |  |  |  |
|  | **Anxiety** | | | | | | |
|  | All psychological interventions | -1.07 (-1.59 to-0.55) I^2^=65.2%, p=0.024 | 4 | 209 |  |  |  |
| **War-affected** | **PTSD symptoms** | | | | | | |
|  | All psychological interventions | -0.44 (-0.61 to -0.27) I^2^=40.7%, p=0.072 | 11 | 1260 |  |  |  |
|  | TF-CBT | -0.51 (-0.74, to -0.28) I^2^=50%, p=0.062 | 6 | 713 |  |  |  |
|  | **Depression** | | | | | | |
|  | All psychological interventions | -0.45 (-0.72 to -0.18) I^2^=70.6%, p=0.001 | 8 | 1060 |  |  |  |
|  | TF-CBT | -0.48 (-0.82 to -0.15) I^2^=74.8%, p=0.001 | 6 | 827 |  |  |  |
|  | **Anxiety** | | | | | | |
|  | All psychological interventions | -0.53 (-1.01 to -0.05) I^2^=91%, p=0.000 | 6 | 1014 |  |  |  |
|  | TF-CBT | -0.64 (-1.18, -0.10) I^2^=90.4%, p=0.000 | 5 | 691 |  |  |  |
| **Refugees** | **PTSD symptoms** | | | | | | |
|  | All psychological interventions | -1.62 (-2.10 to -1.14) I^2^=55.4%, p=0.04 | 7 | 235 | -0.66 (-1.22 to -0.09) I^2^=72.7%, p=0.03 | 3 | 235 |
|  | TF-CBT | -2.12 (-2.71 to -1.53) I^2^=0%, p=0.852 | 3 | 71 | -0.40 (-0.87 to 0.06) I^2^=40.1%, p=0.70 | 2 | 165 |
|  | EMDR | -1.34 (-2.10 to -0.58) I^2^=75%, p=0.953 | 3 | 146 |  |  |  |
|  | **Depression** | | | | | | |
|  | All psychological interventions | -1.27 (-2.04 to -0.50) I^2^=80.5%, p=0.001 | 6 | 195 | -0.73 (-1.57 to 0.10) I^2^=81.3, p=0.02 | 2 | 133 |
|  | TF-CBT | -2.03 (-2.92 to -1.13) I^2^=0%, p=0.953 | 2 | 31 | -0.73 (-1.57 to 0.10) I^2^=81.3%, p=0.02 | 2 | 133 |
|  | EMDR | -0.86 (-2.00 to 0.28) I^2^=89.7%, p=0.001 | 3 | 146 |  |  |  |
| **Domestic violence** | **PTSD symptoms** | | | | | | |
|  | All psychological interventions | -2.04 (-4.02 to -0.07) I^2^=96.5%, p=0.001 | 3 | 210 |  |  |  |
|  | TF-CBT | -2.92 (-3.45 to -2.39) I^2^=0%, p=0.87 | 2 | 117 |  |  |  |
|  | **Depression** | | | | | | |
|  | TF-CBT | -3.24 (-4.40 to -2.09) I^2^=66.6%, p=0.084 | 2 | 117 |  |  |  |

EMDR – eye movement desensitisation and reprocessing therapy, TF-CBT – trauma focused cognitive behavioural therapy, PTSD – post-traumatic stress disorder
