# Supplementary material for: Psychological and pharmacological interventions for posttraumatic stress disorder and comorbid mental health problems following complex traumatic events: Systematic review and component network meta-analysis
Source: PLoS Med. 2020 Aug 19;17(8):e1003262. doi: 10.1371/journal.pmed.1003262 (PMC7446790; doi:10.1371/journal.pmed.1003262)
Supplement: S6 Table — (DOCX) [file pmed.1003262.s008.docx]

S6 Table Mean difference for outcomes by intervention component

| **Intervention components** | **Mean difference versus waitlist (95% CI)** |
| --- | --- |
| Active control | 0.08 (-62.20, 61.67) |
| Placebo | -20.78 (-47.86, 6.35) |
| Support | 3.27 (-21.40, 28.45) |
| Psychoeducation | 3.08 (-15.13, 22.08) |
| Relaxation | 6.78 (-15.36, 28.40) |
| Mindfulness | -5.50 (-29.43, 19.33) |
| Cognitive restructuring | 4.24 (-35.28, 44.24) |
| Virtual reality exposure | 7.81 (-21.34, 36.33) |
| Imaginal exposure | -2.99 (-31.56, 25.82) |
| Multicomponent | -37.95 (-60.84, -15.16) |
| In vivo exposure | 0.70 (-16.69, 19.01) |
| Psychoeducation + Relaxation | -17.41 (-45.69, 12.72) |
| Psychoeducation + cognitive restructuring | 6.69 (-13.90, 61.24) |
| Psychoeducation + imaginal exposure | 10.82 (-14.20, 35.13) |
| Relaxation + mindfulness | 28.02 (-15.08, 70.87) |
| Relaxation + cognitive restructuring | -6.52 (-26.65, 11.95) |
| Relaxation + imaginal exposure | -1.52 (-27.08, 23.01) |
| Mindfulness + Cognitive restructuring | 1.88 (-34.70, 40.08) |
| Cognitive restructuring + in vivo exposure | 0.59 (-34.75, 34.22) |
| Cognitive restructuring + imaginal exposure | -1.88 (-26.76, 23.94) |
| Support + psychoeducation | 5.01 (-36.75, 48.19) |
