# Supplementary material for: Psychological and pharmacological interventions for posttraumatic stress disorder and comorbid mental health problems following complex traumatic events: Systematic review and component network meta-analysis
Source: PLoS Med. 2020 Aug 19;17(8):e1003262. doi: 10.1371/journal.pmed.1003262 (PMC7446790; doi:10.1371/journal.pmed.1003262)
Supplement: Attachment — Submitted filename: Responses to the reviewers.docx [file pmed.1003262.s009.docx]

Responses to the reviewers

| Editors comments | Author responses |
| --- | --- |
| In the last sentence of the Abstract Methods and Findings section, please describe the main limitation(s) of the study's methodology. | We have now included in lines 62-65 a statement about the main methodological limitations of the review. |
| Include a non-technical Author Summary | We have now summarised the rationale, findings and implications of the study using an author summary as per the guidance from PLOS (lines 73-110). |
| The reviewers have raised the issue of whether it's possible to report on any harms (adverse effects) findings of the included studies, or to highlight/elaborate on the absence of this data in the included trials, if relevant - the editors would agree with this point and suggest it be covered in some way depending on what the data can support. | This is an important point and in-keeping with the comments from reviewer 3 we have included a paragraph in the discussion (lines 767-774) that highlights that included studies did not typically report harms data. We have extended this discussion point to draw attention to recommendations about the type of harms data that should be collected, especially for studies of psychological treatments. |
| Reviewers have also queried the inclusion of non-randomized data in the meta-analyses. Obviously it is up to the authors to decide how to address this point but some sensitivity analysis around this issue might be merited - ie to note how the main conclusions might change if only based on randomized trial data (which would be more conventional). | We acknowledge that the inclusion of non-randomised trials in systematic reviews with meta-analyses is not conventional. However our decision to include non-randomised comparisons stemmed from feedback from independent peer review during development of the study protocol. It was felt that our review would be best placed to capture data about emerging and novel therapies and therapies delivered in more pragmatic settings if we included data from non-randomised controlled trials too. We have explained this point in the Methods section (lines 163-165) In the event data from the non-randomised controlled trials formed only a small portion of the totality of evidence that we synthesised and the findings from these studies do no distract from or deviate from the main findings from the randomised controlled trial evidence. Because we did not pool data from randomised and non-randomised trials together it is not possible to undertake sensitivity analysis to discern if findings differ with or without evidence from non-randomised studies. Additionally, we would suggest that it was important to present results from all included studies and as such it is a strength rather than a weakness that we present results from non-randomised and randomised studies. We have included an additional point in the discussion to support this position (lines 722-727). |
| **Reviewer 1 comments** | **Author Responses** |
| Network meta-analysis relies on the assumption of exchangeability.  How was this tested? I did not see it. Lines 260-261 and 267   Please give some detail. | Thanks for the comment. As is common with most NMAs there was insufficient data to test whether there was evidence of inconsistency between direct and indirect evidence. We've added some text (lines 301-308) to clarify:  “The advantages of this approach is that all intervention components can be included in the meta-analyses as long as they form a connected network. An important assumption of the network meta-analysis is consistency between direct (i.e. where trials have specifically compared two or more interventions) and indirect (i.e. data derived from the network where trials have not directly compared interventions) evidence. To assess the validity of this assumption we examined participant and study characteristics and sought input from topic experts. Based on this assessment we judged the data similar enough to combine in the network meta-analysis. However, as is common in most network meta-analyses, there was insufficient data to statistically test this assumption.” |
| Plots: What are the bars around the points? Is that scaled to weight, or what? | We assume you're referring to the forest plots. The bars represent 95% confidence intervals for each study included in the meta-analysis. |
| **Reviewer 2 comments** | **Author responses** |
| Further justifications in including non-randomised trials in this SR is indicated - given there are sufficient data from RCTs as evidenced in the meta-analyses and NMA, the value of non-randomised trials is doubtful. Including effect sizes from a single non-randomised trials in the results of secondary outcomes and subgroup analyses, in my view, does not add to the evidence. | We accept that our review includes substantial data from RCTs but as discussed in response to the editor’s points, we included non-randomised studies following feedback from independent peer review while the protocol was being developed. It was agreed with the steering committee that we should aim to include data from non-randomised controlled trials as there was a chance these types of studies might have tested novel and emerging therapies as well as being of a more pragmatic nature. This rationale is provided now in the Methods section (lines 163-165) Ultimately we were able to draw on an extensive dataset of RCTs which eclipsed findings from non-randomised studies and we have explained this in the discussion (lines 722-727). Overall we consider it to be an important step to include all available data as per the protocol including that from non-randomised trials. The inclusion of these studies does not detract from the main findings, but rather reaffirms them. |
| In reporting the results, please provide an overview of the studies and the interventions investigated. It is of essence to understand the nature and intensity of the interventions, e.g. clinical settings, programme comprising xx sessions? range of treatment duration (e.g. over 6 months), time/dose of cumulative sessions, .... etc. Same applies to the pharmacological interventions. Such a summary description will illustrate the intervention intensity and design better for the readers to interpret the results of their effectiveness. It will also set the scene for the discussion arguing phasing being an element of multi-component interventions targeting a range of symptoms in addition to core PTSD symptoms in this population. | Thank you for making this point. We agree that is a useful and important step to include descriptive details about the clinical settings of the studies as well as detail about the duration, frequency and length of treatment/dosing for both psychological and pharmacological interventions. We have now included in a revised Results section (lines 347-352) details about setting for all the included studies and have reported (lines 372-387), where data permitted, mean data about treatment duration, frequency, and length/dose. |
| In reporting the results, it is preferable for all the statistically significant MAs to be written out in full, i.e. k=xx, n=xxx, SMD, 95% CI, p value, I squared, as they help the readers to understand the effects the authors report through texts and to interpret the effects in the context of the available evidence. Also in reporting the primary analysis pulling all psychological interventions comparing with all controls across all populations, think it is better that the results are reported separately for post-treatment (plus the point above re good to know how long treatment lasted on average), and at other follow-up time points (think only 6-months). | This is a useful suggestion that has helped us to unpick the reporting of the primary outcome and to make reporting of meta-analyses clearer. We have now broken up reporting of the primary outcome by post-treatment and 6-month outcomes as suggested. And we have now included study characteristics data (k=xx; n=xx) to the meta-analyses reporting in the text for significant results (lines 409-448). This format has also been used to report the findings from the network meta-analysis (lines 611-622). The priority here is to report results in full in the text for findings that are not reported in tables or the forest plots. All data about number of studies and number of participants per meta-analysis, along with effect sizes, confidence intervals and significance tests are given in tables S2 and S3. |
| In terms of limitations, I would suggest commenting on the lack of medium or long-term follow up data. | This is an important observation and we have now included in the limitations section a brief discussion (lines 760-766) about the merits of collecting outcome data over the long term and put this issue in the context of complex trauma. |
| Please check through the numbers of excluded full text in the PRISMA flowchart: the numbers do not add up to 402. | We apologise for the error in reporting the study flow in the PRISMA flowchart. The figure had not updated following the latest search. This has now been corrected. |
| Please also check through the captions for Figures 4-6 | Thank you for spotting the labelling errors with these figures which have been corrected. |
| **Reviewer 3 comments** | **Author responses** |
| I couldn't find the results on harms (worsening of PTSD) | The reviewer raises an important topic for discussion. Harms data were not routinely reported across the included studies making it difficult to present meaningful data on such events for both psychological and pharmacological trials. We think this is a key point that we have addressed in a revised discussion section (lines 767-774) where we flag up the need to collect a range of harms data using mixed-methods to better understand harms in complex trauma treatments. |
| I was struck by the few studies in the meta-analysis that had measured quality of life and the lack of statistically significant improvement in those few studies that had measured it. Considering the movement to broaden the conceptualisation of, and include patient perspectives on, outcomes (the recovery movement), would the authors consider making some kind of statement about quality of life results in the discussion? | This point provides a welcome opportunity for us to emphasise the importance of collecting data on more global and patient centred outcomes in future research. We agree with the reviewer that this is a topic that merits further discussion and we have revised our discussion to reflect this (lines 711-720). |
